# The CD147 expression in CD4^+^ T cells is a novel biomarker for predicting efficacy of IL‐17A inhibitor and psoriasis recurrence

**DOI:** 10.1002/ctm2.1568

**Published:** 2024-02-01

**Authors:** Panpan Liu, Lei Li, Yehong Kuang, Wu Zhu, Jie Li, Xiang Chen, Cong Peng

**Affiliations:** ^1^ Department of Dermatology Xiangya Hospital Central South University Changsha Hunan China; ^2^ Furong Laboratory Changsha Hunan China; ^3^ Hunan Key Laboratory of Skin Cancer and Psoriasis Changsha Hunan China; ^4^ National Engineering Research Center of Personalized Diagnostic and Therapeutic Technology Changsha Hunan China; ^5^ National Clinical Research Center for Geriatric Disorders Xiangya Hospital Central South University Changsha Hunan China

Dear Editor,

Here, we demonstrated the crucial functions of CD147 expressed by CD4^+^ T cells in the pathogenesis and recurrence of psoriasis and developed two novel predictive models that accurately predict the therapeutic effect and durable efficacy of IL‐17A inhibitor, providing an understanding of psoriasis pathogenesis and implications for personalised treatment approaches in psoriasis therapy.

Although significant progress had been made in the treatment of psoriasis, the lack of effective biomarkers for predicting treatment efficacy and psoriasis recurrence remains a big challenge.^1^, ^2^ CD147 is a transmembrane glycoprotein that was reported to be highly expressed in psoriatic lesions.^3^ However, the role of CD147 expressed by CD4^+^ T cells in the progression of psoriasis remains uncertain. We found after IL‐17A inhibitor treatment, psoriasis patients with a psoriasis area and severity index (PASI) 75 response showed an elevated CD147 expression on CD4^+^ T cells (Table S1 and Figures 1A and S1A,B). However, psoriasis patients without a PASI75 response showed a decreased CD147 expression on CD4^+^ T cells (Table S1 and Figures 1B and S1C). The improvements in the PASI score were positively correlated with the changes in the CD147 expression of psoriasis patients following IL‐17A inhibitor therapy (Figure 1C), whereas negatively associated with the CD147 expression of psoriasis patients before treatment (Figure 1D). Furthermore, following 4 weeks of IL‐17A inhibitor treatment or after 1 year of withdrawal, psoriasis patients with recurrence showed less variation in CD147 expression on the CD4^+^ T cells than those without recurrence (Figure 1E,F and Table S2). Two innovative predictive models had been developed based on the discovery of elevated CD147 expression by CD4^+^ T cells of psoriasis patients who showed positive treatment response and sustained remission. These models effectively forecast the therapeutic efficacy (Figures 1G and S2A–D and Tables S3 and S4) and long‐term effectiveness of IL‐17A inhibitors for treating psoriasis (Figures 1H and S2E–H and Tables S5 and S6). Researchers found that lipopolysaccharide‐induced NF‐κBp65 phosphorylation was a predictive biomarker of nonresponse to adalimumab.^4^ Here, we developed two easy‐to‐apply models with the decent predictive capability, which provided suggestions for individualised treatment plans and patient education. Considering the limitations of the study, multicentre studies and external validation were needed to improve the prediction model.

**FIGURE 1:**
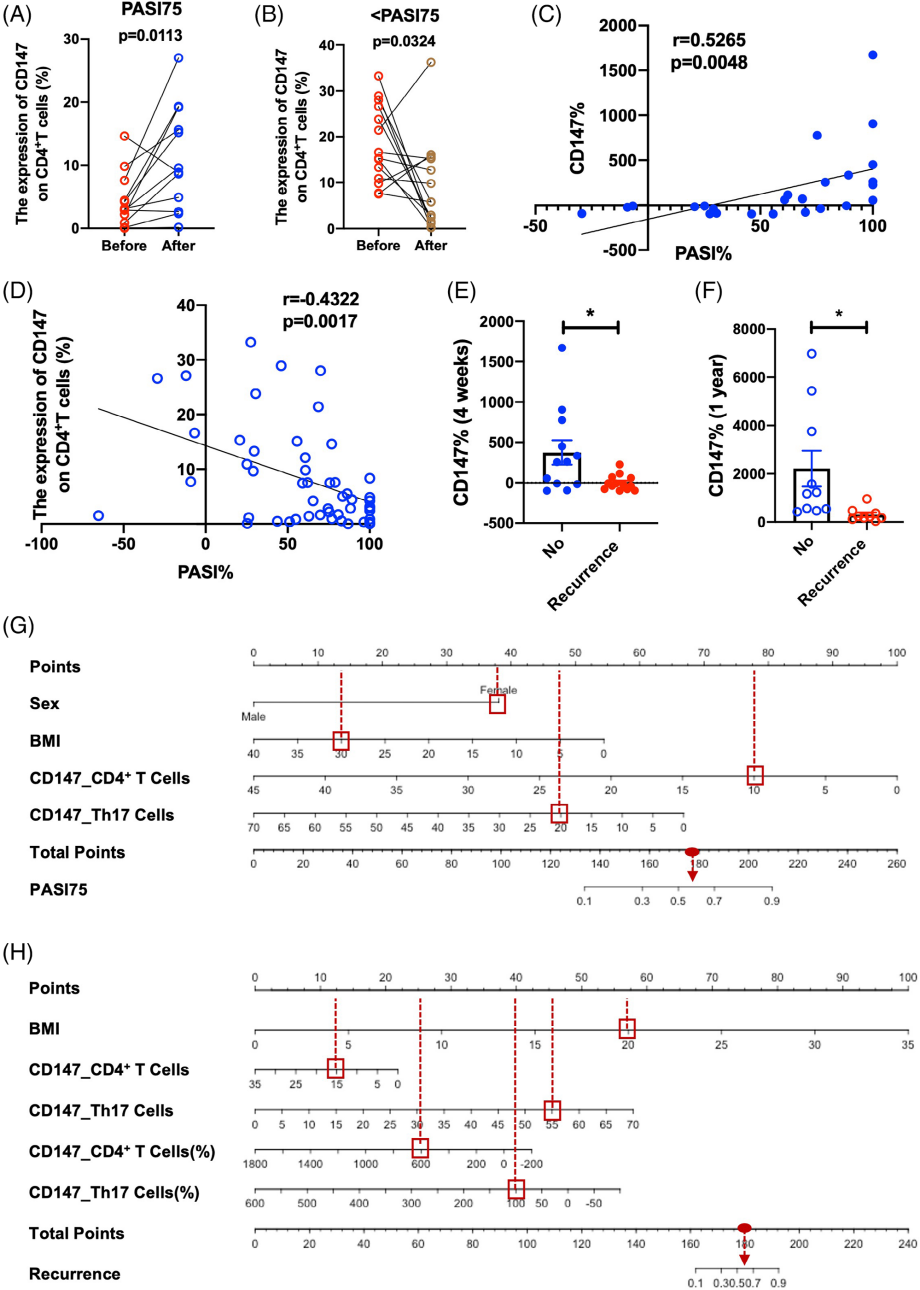
CD147 expression on CD4^+^ T cells is highly correlated with the treatment efficacy and psoriasis recurrence. (A) The CD147 expression on the CD4^+^ T cells increased in the peripheral blood mononuclear cells (PBMCs) of psoriasis patients achieved a PASI75 response after the treatment of IL‐17A inhibitor. Psoriasis patients were treated with IL‐17A inhibitor for 4 weeks. The expression of CD147 on the CD4^+^ T cells in the PBMCs of psoriasis patients achieved a PASI75 response (*n* = 12) was detected by flow analysis. (B) The CD147 expression on the CD4^+^ T cells decreased in the PBMCs of psoriasis patients without a PASI75 response after the treatment of IL‐17A inhibitor. Psoriasis patients were treated with IL‐17A inhibitor for 4 weeks. The CD147 expression on the CD4^+^ T cells in the PBMCs of psoriasis patients without a PASI75 response (*n* = 14) was detected by flow analysis. (C) The changes in the expression of CD147 on the CD4^+^ T cells after the treatment of IL‐17A inhibitor were positively correlated with the improvement of PASI score (*n* = 26). (D) The expression of CD147 on the CD4^+^ T cells before the treatment of IL‐17A inhibitor were negatively correlated with the improvement of PASI score (*n* = 50). (E) The changes in the expression of CD147 on the CD4^+^ T cells of psoriasis patients with recurrence (recurrence, *n* = 14) were lower than that of patients without recurrence (no, *n* = 12). Psoriasis patients were treated with IL‐17A inhibitor for 4 weeks and the changes in the expression of CD147 on the CD4^+^ T cells was detected by flow analysis. (F) The changes in the expression of CD147 on the CD4^+^ T cells of psoriasis patients with recurrence (recurrence, *n* = 9) were lower than that of patients without recurrence (no, *n* = 10). Psoriasis patients were treated with IL‐17A inhibitor for 1 year and the changes in the expression of CD147 on the CD4^+^ T cells was detected by flow analysis. (G) The predictive nomogram of the PASI75 efficacy of IL‐17A inhibitor for the treatment of psoriasis. CD147_CD4^+^ T cells: the expression of CD147 on CD4^+^ T cells. CD147_Th17 cells: the expression of CD147 on Th17 cells. (H) The predictive nomogram of the risk of recurrence of psoriasis. CD147_CD4^+^ T cells: the expression of CD147 on CD4^+^ T cells before treatment, CD147_Th17 cells: the expression of CD147 on Th17 cells before treatment, CD147_CD4^+^ T cells (%): the changes in the expression of CD147 on CD4^+^ T cells after the treatment of IL‐17A inhibitor for 4 weeks, CD147_Th17 cells (%): the changes in the expression of CD147 on the Th17 cells after the treatment of IL‐17A inhibitor for 4 weeks. The use of predictive models is shown in the supplemental materials. Data are presented as the mean ± standard error of the mean (SEM). ^*^ *p* < .05.

To investigate the role of CD147 in psoriasis, CD4^+^ T‐cell‐specific CD147‐knockout mice (CD4*^cre^* Bsg*^fl/fl^*) was developed to establish the psoriasis‐like model. We found genomic knock out CD147 in CD4^+^ T cells aggravated the pathogenesis of psoriasis (Figures 2A, S3A–D and S4A–E). The number of Th17 and Th1 cells in skin lesions of CD4*^cre^* Bsg*^fl/fl^* group was notably elevated when compared to Bsg*^fl/fl^* group (Figures 2B,C, S3E–H and S4F–I).

**FIGURE 2:**
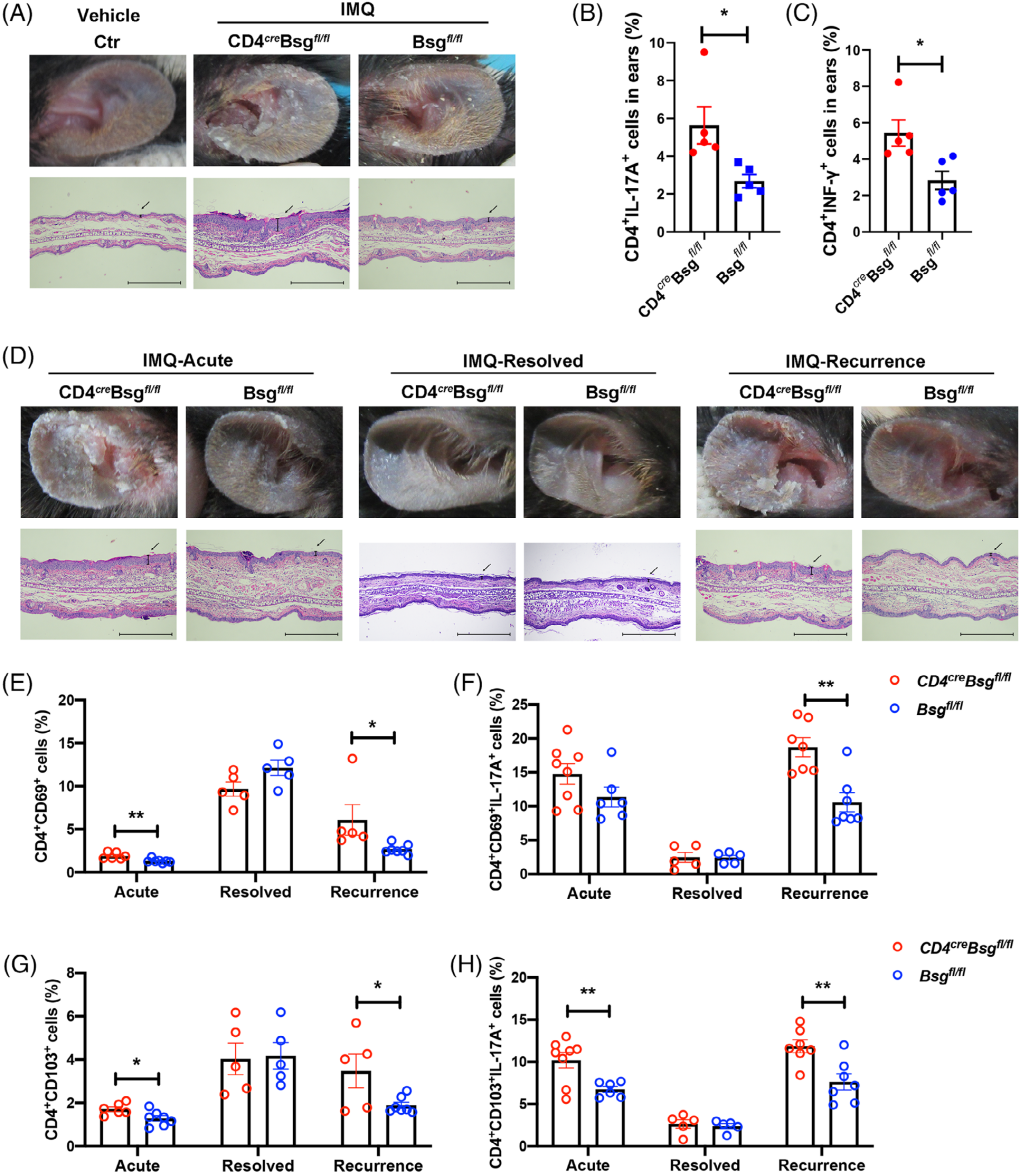
Genomic knock out CD147 in CD4^+^ T cells aggravates the pathogenesis of psoriasis and facilitates the recurrence of psoriasis. (A–C) CD147 deficiency in CD4^+^ T cells aggravate the imiquimod (IMQ)‐induced psoriasis‐like skin phenotype. IMQ was applied for the CD4*^cre^* Bsg*^fl/fl^* and Bsg*^fl/fl^* mice ears (*n* = 5). (A) Phenotypic manifestation and haematoxylin and eosin (H&E) staining of Control (Ctr), CD4*^cre^* Bsg*^fl/fl^* and Bsg*^fl/fl^*. Scale bars, 100 μm. Arrows indicate the changes in epidermal thickness. (B and C) Cell suspensions from the ear skin were analysed by using flow cytometry. (B) Th17 (CD4^+^IL‐17A^+^) cells. (C) Th1 (CD4^+^IFN‐γ^+^) cells. (D–H) IMQ was applied for the CD4*^cre^* Bsg*^fl/fl^* and Bsg*^fl/fl^* mice ears to established psoriasis‐like recurrence mouse model as shown in Figure S5 (*n* = 5–8). (D) Phenotypic manifestation and H&E staining of mice in acute, resolved, and recurrence groups (*n* = 5–8). Scale bars, 100 μm. Arrows indicate the changes in epidermal thickness. (E–H) Cell suspensions from the ear skin were analysed by using flow cytometry. (E) CD4^+^ CD69^+^ T_RM_ cells in acute, resolved and recurrence groups (*n* = 5–8). (F) The expression of IL‐17A in CD4^+^ CD69^+^ T_RM_ cells in acute, resolved and recurrence groups (*n* = 5–8). (G) CD4^+^ CD103^+^ T_RM_ cells in acute, resolved and recurrence groups (*n* = 5–8). (H) The expression of IL‐17A in CD4^+^ CD103^+^ T_RM_ cells in acute, resolved and recurrence groups (*n* = 5–8). Data are presented as the mean ± SEM. ^*^ *p* < .05, ^**^ *p* < .01.

Skin‐resident memory T cells (T_RM_) are crucial for preserving immunological memory and mediating rapid immune responses against recurring pathogens. T_RM_ cells express CD69 or CD103,^5^ and contribute to the chronic inflammation by continuously producing proinflammatory cytokines, such as IL‐17A.^6^ We found genomic knock out CD147 in CD4^+^ T cells accelerated the recurrence progression of psoriasis (Figures 2D, S5 and S6). We noted that during the acute stage, the number of CD4^+^CD103^+^ T_RM_ cells increased in CD4*^cre^* Bsg*^fl/fl^* mice with higher expression of IL‐17A (Figures 2E–H and S7A,B). Nevertheless, there was no notable difference in the quantity of T_RM_ cells and its IL‐17A expression in the CD4*^cre^* Bsg*^fl/fl^* and Bsg*^fl/fl^* groups during the resolved stage (Figures 2E–H and S7C,D). During the recurrence stage, the number of CD4^+^CD69^+^ and CD4^+^CD103^+^ T_RM_ cells and its IL‐17A expression were notable elevated in CD4*^cre^* Bsg*^fl/fl^* mice compared with Bsg*^fl/fl^* mice (Figures 2E–H and S7E,F). Taken together, CD147 deficiency increased the number of T_RM_ cells in skin lesions with a higher IL‐17A expression, accelerating the recurrence of psoriasis.

In psoriasis, the expression of CD147 was negatively corelated to the IL‐17A expression in CD4^+^ T cells (Figures 3A and S8A,B and Table S1). We then explored the function of CD147 on the differentiation of Th17 cells. The findings showed that CD147 deficiency promoted the differentiation of pathogenic Th17 cells (Figures 3B and S8C). RNA sequencing indicated that the impact of CD147 on the polarisation of Th17 cells involves the cytokine–receptor interaction pathway, such as IL‐6–STAT3 signalling pathways (Figure S8D). And we found CD147 effectively inhibited the activation of p‐JAK2 and p‐STAT3 in CD4^+^ T cells (Figures 3C and S8E,F), which is the key transcription factor of IL‐17A.^7^ Since CD147 could modify intracellular signalling by interacting with various proteins,^8^ we tested the interaction between CD147 and IL‐6R and showed that CD147 deficiency in CD4^+^ T cells enhanced the IL‐6R expression (Figures 3D,E and S8G). The endogenous and exogenous immunoprecipitations revealed that CD147 could interact with IL‐6R (Figures 3F,G and S8H). Confocal immunofluorescence showed that CD147 interacted with IL‐6R on the membrane of CD4^+^ T cells before the stimulation with IL‐6. When IL‐6 was applied, the CD147–IL‐6R complex was endocytosed into the lysosome (Figure 3H). Therefore, CD147 promoted the endocytosis of IL‐6R and then inhibited Th17 differentiation.

**FIGURE 3:**
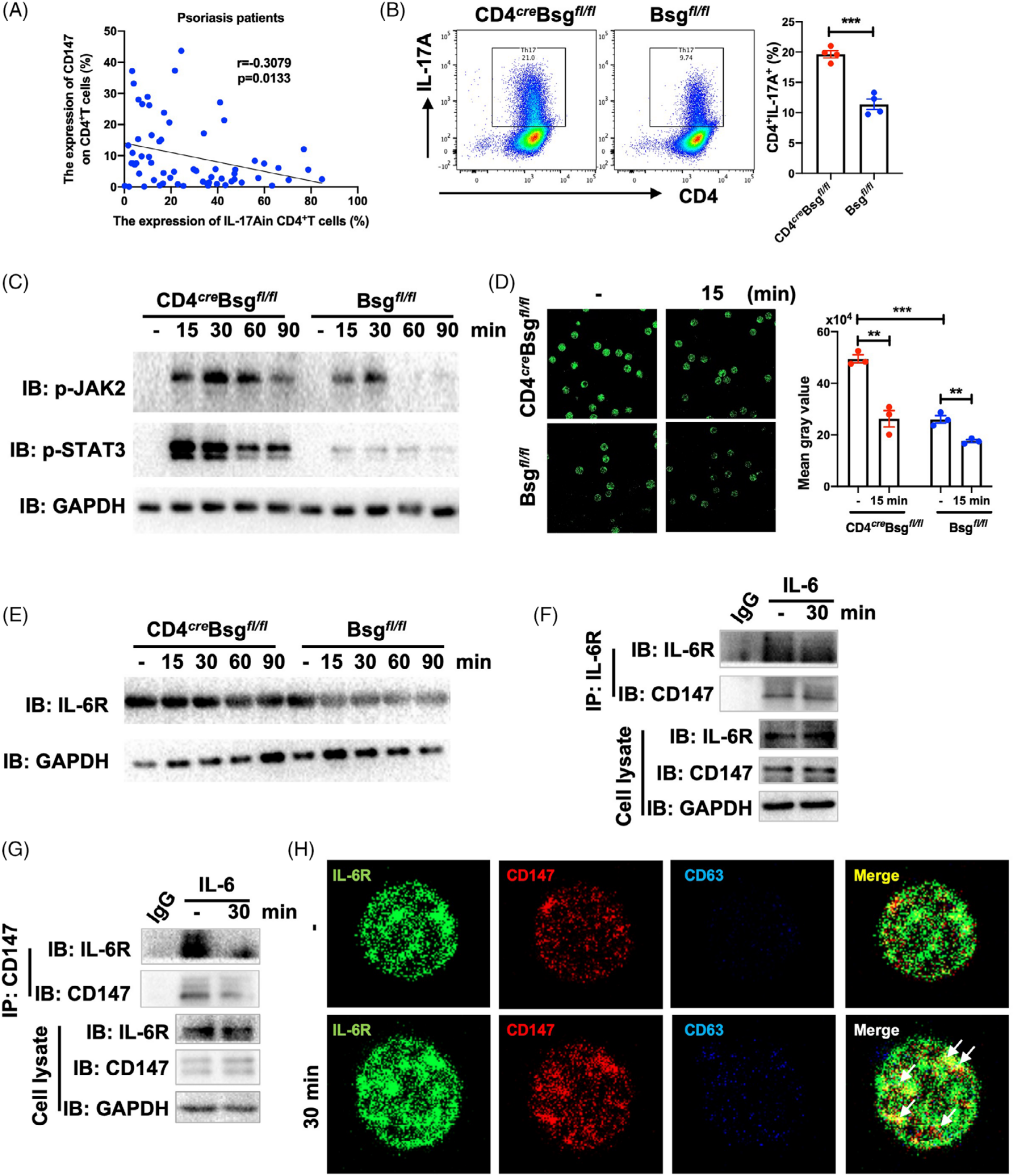
CD147 expression in CD4^+^ T cells attenuates Th17 skewing through the endocytosis of IL‐6R. (A) The correlation analysis of the expression of CD147 and IL‐17A in CD4^+^ T cells in peripheral blood mononuclear cells (PBMCs) of psoriasis patients (*n* = 64). (B) CD147 inhibits the differentiation of Th17 cells. Representative flow cytometry plots (left). Statistical data of Th17 (CD4^+^IL‐17A^+^) cells (*n* = 4). (C) CD147 inhibits the activation of JAK2–STAT3 signaling pathway in CD4^+^ T cells. Mouse CD4^+^ T cells were obtained from the spleen of CD4*^cre^* Bsg*^fl/fl^* and Bsg*^fl/fl^* mice and activated in vitro with IL‐6 (30 ng/mL) for 15, 30, 60 or 90 min. (D) CD147 deficiency in CD4^+^ T cells enhances the expression of IL‐6R. CD4^+^ T cells obtained from the spleen of CD4*^cre^* Bsg*^fl/fl^* and Bsg*^fl/fl^* mice and activated in vitro with IL‐6 (30 ng/mL) for 15 min. Representative figures of immunofluorescence staining (left). Statistical data of the expression of IL‐6R on CD4^+^ T cells (right). (E) CD147 deficiency in CD4^+^ T cells maintains the expression of IL‐6R. CD4^+^ T cells obtained from the spleen of CD4*^cre^* Bsg*^fl/fl^* and Bsg*^fl/fl^* mice and activated in vitro with IL‐6 (30 ng/mL) for 15, 30, 60 or 90 min. (F and G) CD147 interacts with IL‐6R on CD4^+^ T cells. (F) Cells extracts were utilised for immunoprecipitation using an IL‐6R antibody or control immunoglobulin G (IgG). (G) Cells extracts were utilised for immunoprecipitation using a CD147 antibody or control IgG. (H) CD147 promotes the endocytosis of IL‐6R on CD4^+^ T cells. CD4^+^ T cells obtained from the spleen of wild‐type (WT) mice and activated in vitro with IL‐6 for 30 min. The CD4^+^ T were then fixed and stained using anti‐IL‐6R (green), anti‐CD147 (red) and anti‐CD63 (blue) fluorescence‐conjugated antibody. Colocalisation of IL‐6R, CD147 and CD63 was performed using confocal microscopy. The yellow indicated the colocalisation of IL‐6R and CD147. The white indicates the endocytosis of IL‐6R–CD147 complex. Data are presented as the mean ± SEM. ^**^ *p* < .01, ^***^ *p* < .001.

The retention of T_RM_ cells was dependent on the IL‐15R expression on the membrane.^9^ Thus, we investigated whether there was an interaction between CD147 and IL‐15R. The findings indicated that IL‐15R protein expression was notably reduced on Bsg*^fl/fl^* CD4^+^ T cells, while no notable variation in the IL‐15R mRNA expression was found in CD4*^cre^* Bsg*^fl/fl^* and Bsg*^fl/fl^* CD4^+^ T cells, both before and after IL‐15 stimulation (Figure 4A–C). We further showed that CD147 interacted with IL‐15R in CD4^+^ T cells by endogenous immunoprecipitation (Figure 4D,E). Confocal immunofluorescence revealed that CD147 interacted with IL‐15R on the membrane of CD4^+^ T cells. Following the IL‐15 simulation, the CD147–IL‐15R complex was endocytosed into the lysosome (Figure 4F). In addition, CD147 deficiency promoted the expression of IL‐17A in T_RM_ cells (Figure 4G).

**FIGURE 4:**
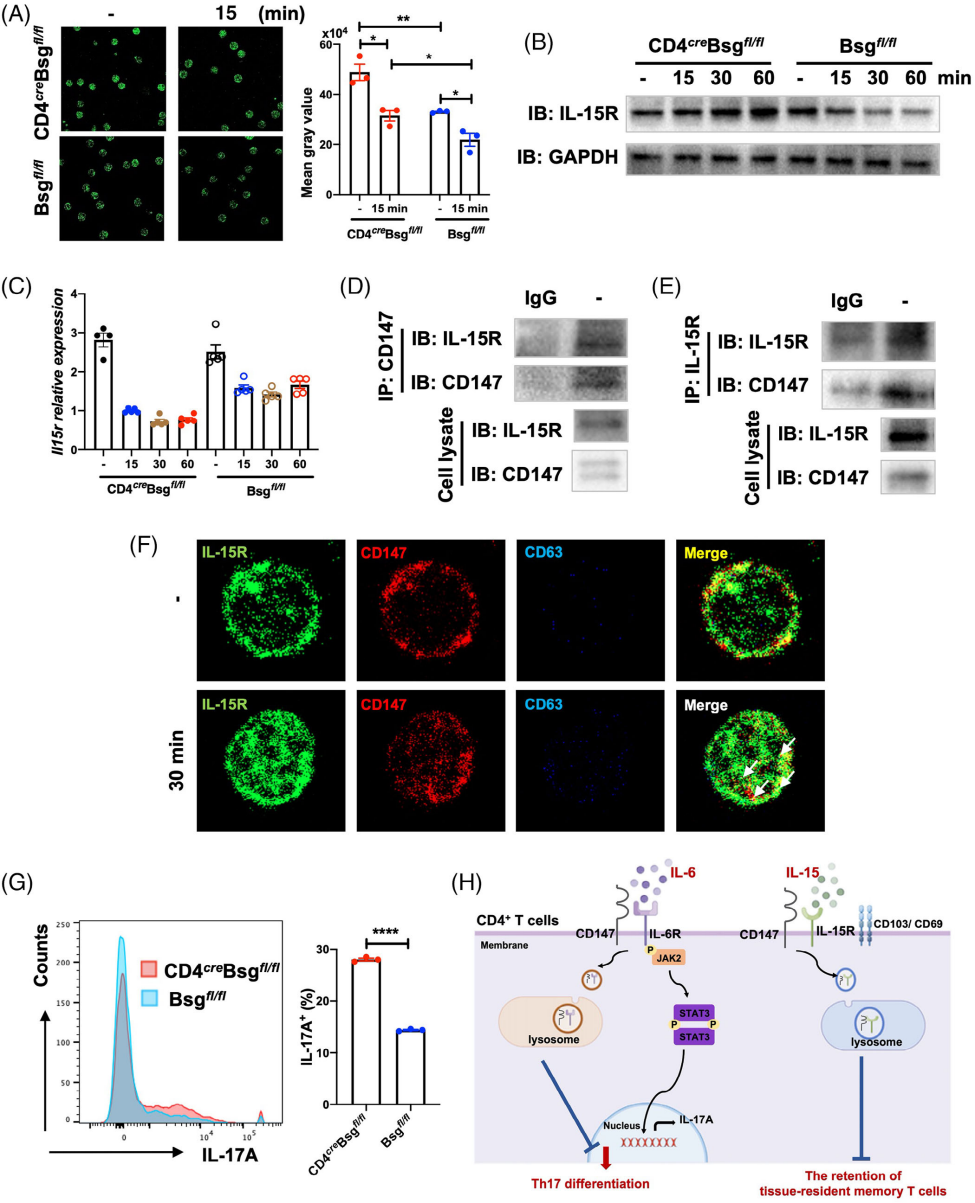
CD147 interacts with IL‐15 receptor and induces its endocytosis on CD4^+^ T cells. (A) CD147 deficiency in CD4^+^ T cells enhances the expression of IL‐15R. CD4^+^ T cells obtained from the spleen of CD4*^cre^* Bsg*^fl/fl^* and Bsg*^fl/fl^* mice and activated in vitro with IL‐15 for 15 min. Immunofluorescence staining of IL‐15R (green) on paraformaldehyde‐fixed CD4^+^ T cells. Representative figures of immunofluorescence staining (left). Statistical data of the expression of IL‐15R on CD4^+^ T cells (right). (B) CD147 deficiency in CD4^+^ T cells maintains the expression of IL‐15R. CD4^+^ T cells obtained from the spleen of CD4*^cre^* Bsg*^fl/fl^* and Bsg*^fl/fl^* mice and activated in vitro with IL‐15 for 15, 30 or 60 min. (C) The effect of CD147 on the mRNA expression of *Il15r* in CD4^+^ T cells. CD4^+^ T cells obtained from the spleen of CD4*^cre^* Bsg*^fl/fl^* and Bsg*^fl/fl^* mice and activated in vitro with IL‐15 for 15, 30 or 60 min. (D and E) CD147 interacts with IL‐15R on CD4^+^ T cells. CD4^+^ T cells obtained from the spleen of WT mice. (D) Cells extracts were utilised for immunoprecipitation using an CD147 antibody or control IgG. (E) Cells extracts were utilised for immunoprecipitation using an IL‐15R antibody or control IgG. (F) CD147 promotes the endocytosis of IL‐15R on CD4^+^ T cells. CD4^+^ T cells obtained from the spleen of WT mice and activated in vitro with IL‐15 for 30 min. The CD4^+^ T were then fixed and stained using anti‐IL‐15R (green), anti‐CD147 (red) and anti‐CD63 (blue) fluorescence‐conjugated antibody. Colocalisation of IL‐15R, CD147 and CD63 was performed using confocal microscopy. The yellow indicated the colocalisation of IL‐15R and CD147. The white indicates the endocytosis of IL‐15R–CD147 complex. (G) CD147 inhibits the expression of IL‐17A in T_RM_ cells. CD4^+^CD103^+^ T_RM_ cells were obtained from the spleen of WT mice and activated in vitro under IL‐6 conditions. Representative flow cytometry plots (left). Flow cytometric statistical data of the expression of IL‐17A in T_RM_ cells (*n* = 3). (H) The mechanism of CD147 inhibits the Th17 differentiation and the retention of T_RM_ cells. Data are presented as the mean ± SEM. ^*^ *p* < .05, ^**^ *p* < .01, ^****^ *p* < .0001.

In summary, we found CD147 expression by CD4^+^ T cells was correlated with the treatment efficacy and recurrence of psoriasis. CD147 expression by CD4^+^ T cells may serve as a predictive marker for the efficacy and recurrence of IL‐17A inhibitors treatment of psoriasis. Moreover, CD147 plays a protective function in the development and recurrence of psoriasis by inhibiting the Th17 differentiation and the retention of T_RM_ cells (Figure 4H).

## AUTHOR CONTRIBUTIONS

Panpan Liu, Xiang Chen and Cong Peng conceived the project and wrote the manuscript. Panpan Liu designed, performed experiments, and analysed the data. Lei Li, Yehong Kuang, and Jie Li helped with mouse experiments. Wu Zhu, Yehong Kuang, and Jie Li diagnosed with plaque psoriasis and provided patient samples and information. Panpan Liu and Cong Peng edited the manuscript. Xiang Chen and Cong Peng supervised the work.

## CONFLICT OF INTEREST STATEMENT

The authors declare they have no conflicts of interest.

## ETHICS STATEMENT

The study was approved by the Ethics Committees of Xiangya Hospital (2019030227 and 202103587).

## Supporting information

Supporting Information

## Data Availability

More information about resources should be contacted to the lead contact, Cong Peng (pengcongxy@csu.edu.cn). The number for the sequencing data is PRJNA933282. Source data are provided in this paper.

## References

[1] Ghoreschi K , Balato A , Enerback C , Sabat R . Therapeutics targeting the IL‐23 and IL‐17 pathway in psoriasis. Lancet. 2021;397:754‐766. doi:10.1016/S0140‐6736(21)00184‐7 33515492 10.1016/S0140-6736(21)00184-7

[2] Armstrong AW , Read C . Pathophysiology, clinical presentation, and treatment of psoriasis: a review. JAMA. 2020;323:1945‐1960. doi:10.1001/jama.2020.4006 32427307 10.1001/jama.2020.4006

[3] Wu LS , Li F‐F , Sun L‐D , et al. A miRNA‐492 binding‐site polymorphism in BSG (basigin) confers risk to psoriasis in central south Chinese population. Hum Genet. 2011;130:749‐757. doi:10.1007/s00439‐011‐1026‐5 21655935 10.1007/s00439-011-1026-5

[4] Andres‐Ejarque R , Ale HB , Grys K , et al. Enhanced NF‐kappaB signaling in type‐2 dendritic cells at baseline predicts non‐response to adalimumab in psoriasis. Nat Commun. 2021;12:4741. doi:10.1038/s41467‐021‐25066‐9 34362923 10.1038/s41467-021-25066-9 PMC8346545

[5] Park CO , Fu X , Jiang X , et al. Staged development of long‐lived T‐cell receptor alphabeta T(H)17 resident memory T‐cell population to *Candida albicans* after skin infection. J Allergy Clin Immunol. 2018;142:647‐662. doi:10.1016/j.jaci.2017.09.042 29128674 10.1016/j.jaci.2017.09.042 PMC5943196

[6] Chen L , Shen Z . Tissue‐resident memory T cells and their biological characteristics in the recurrence of inflammatory skin disorders. Cell Mol Immunol. 2020;17:64‐75. doi:10.1038/s41423‐019‐0291‐4 31595056 10.1038/s41423-019-0291-4 PMC6952397

[7] Shen H , Chen ZW . The crucial roles of Th17‐related cytokines/signal pathways in *M. tuberculosis* infection. Cell Mol Immunol. 2018;15:216‐225. doi:10.1038/cmi.2017.128 29176747 10.1038/cmi.2017.128 PMC5843620

[8] Zhang X , Guo Y , Xiao T , et al. CD147 mediates epidermal malignant transformation through the RSK2/AP‐1 pathway. J Exp Clin Cancer Res. 2022;41:246. doi:10.1186/s13046‐022‐02427‐w 35964097 10.1186/s13046-022-02427-w PMC9375950

[9] Mami‐Chouaib F , Blanc C , Corgnac S , et al. Resident memory T cells, critical components in tumor immunology. J Immunother Cancer. 2018;6:87. doi:10.1186/s40425‐018‐0399‐6 30180905 10.1186/s40425-018-0399-6 PMC6122734

