# Supplementary material for: The CD147 expression in CD4+ T cells is a novel biomarker for predicting efficacy of IL‐17A inhibitor and psoriasis recurrence
Source: Clin Transl Med. 2024 Feb 1;14(2):e1568. doi: 10.1002/ctm2.1568 (PMC10835189; doi:10.1002/ctm2.1568)
Supplement: Supplementary file 1 — Supporting Information [file CTM2-14-e1568-s001.docx]

**Supplementary materials**

**The CD147 expression in CD4^+^ T cells is a novel biomarker for predicting efficacy of IL-17A inhibitor and psoriasis recurrence**

**List of Supplementary Information**

**Materials and Methods**

**Figure S1. CD147 expression on CD4^+^ T cells is highly correlated with the treatment efficacy and disease recurrence**

**Figure S2. The nomogram predicting the efficacy of IL-17A inhibitor and the risk of psoriasis recurrence based on CD147 expression on CD4^+^ T cells**

**Figure S3. Genomic knock out CD147 in CD4^+^ T cells aggravates IMQ-induced psoriasis-like dermatitis**

**Figure S4. Genomic knock out CD147 in CD4^+^ T cells aggravates IL-23-induced psoriasis-like dermatitis**

**Figure S5. IMQ-induced psoriasis-like recurrence mouse model**

**Figure S6. CD147 deficiency in CD4^+^ T cells facilitates the recurrence of psoriasis**

**Figure S7. CD147 deficiency in CD4^+^ T cells facilitates the infiltration of T_RM_ in skin lesions**

**Figure S8. CD147 expression in CD4^+^ T cells attenuates Th17 skewing through the endocytosis of IL-6R**

**Supplementary Table 1. Demographics and clinical characteristics of the psoriasis patients**

**Supplementary Table 2. Demographics and clinical characteristics of the psoriasis patients treated with IL-17A inhibitor**

**Supplementary Table 3. Differences between demographic and clinical characteristics of psoriasis patients with or without PASI75**

**Supplementary Table 4. The univariate logistic regression analysis of psoriatic patients in cohort 1**

**Supplementary Table 5. Differences between demographic and clinical characteristics of psoriasis patients with or without recurrence**

**Supplementary Table 6. The univariate logistic regression analysis of psoriatic patients in cohort 2**

**Materials and Methods**

**Human subjects**

This study included plaque psoriasis. Plaque psoriasis was diagnosed by either evaluating the clinical presentation or examining the histopathology. The severity of the disease was evaluated using psoriasis area and severity index (PASI) scores (*1*). The Table S1 and S2 display the details of the patient information. All psoriasis patients were treated with IL-17A inhibitor (Ixekizumab or Secukinumab). Peripheral blood was collected from psoriasis patients at the time of before treatment, 4 weeks after treatment, and 1 year after treatment. PASI75 defined as 75% improvement in PASI score (*2*). The recurrence of psoriasis defined as the appearance of the new psoriatic lesions or the total PASI score increased to 50% of the original PASI (*3*).The changes in the expression of CD147 on the CD4^+^ T cells (CD147%) = (the expression of CD147 on the CD4^+^ T cells after the treatment of IL-17A inhibitor for 4 weeks - the expression of CD147 on the CD4^+^ T cells before IL-17A inhibitor treatment)/the expression of CD147 on the CD4^+^ T cells before IL-17A inhibitor treatment. The improvement of PASI score (PASI%) = (the PASI score before IL-17A inhibitor treatment - the PASI score after IL-17A inhibitor treatment for 4 weeks)/the PASI score before IL-17A inhibitor treatment. Psoriasis patients without recurrence (No): psoriasis patients did not recurrence within 1 year of standardized treatment with IL-17A inhibitor. Psoriasis patients with recurrence (Recurrence): psoriasis patients recurred within 1 year of standardized treatment with IL-17A inhibitor. All experiments involving clinical samples have been guaranteed to have an assessment of the outcome without bias. Xiangya hospital granted ethical approval. All patients and healthy donors provided their informed consent.

**Flow cytometry**

We collected the peripheral blood from psoriasis patients and healthy controls and obtained the PBMCs using lymphocyte isolation fluid. We obtained the single-cell suspensions from the ears skin and spleen of mice. Lysing Buffer was used to remove red blood cells (BD Cat. 555899). Incubation of skin lesions at 37°C for 60 min with collagenase IV (Sigma-Aldrich Cat. V900893) and dispase II (Sigma-Aldrich Cat. D4693). Cells were filtered through a 40-micron mesh strainer (BD Cat. 352340) and then were stained with fluorophore-conjugated antibodies. Live/dead stain (BioLegend Cat. 432102) and Fc block (BioLegend Cat. 101302) were used to stain the cells. And then, cells were stained with antibodies specific for CD4 (BioLegend Cat. 300532) and CD147 (BioLegend Cat. 306207), or CD45 (BioLegend Cat. 103131), CD4 (BioLegend Cat. 100443), CD69 (BioLegend Cat. 104526), and CD103 (BioLegend Cat. 121441). For intracellular cytokine staining, cells were stimulated for 5 hours with PMA (10 ng/ml; Sigma Cat. 79346), ionomycin (1 mg/ml; TOCRIS Cat. 56092-8-0), and GolgiPlug (1000×; BD Cat. 555029). For intracellular and intranuclear staining, cells were fixed and permeabilized using the Staining Buffer Kit (eBioscience Cat. 00-5523-00). Cells were stained intracellularly with antibodies specific for IL-17A (BioLegend Cat. 512328), IFN-γ (BioLegend Cat. 505815), or IL-17A (BioLegend Cat. 506915). All data were acquired on a Cytek Dxp Athena or FACS LSRFortessa. And flowJo software was used to evaluate the acquired data. Single, live cells were gated for analysis of stained populations.

**Generation of transgenic mice specifically deficiency CD147 in CD4^+^ T cells**

Experiments were conducted using six to eight weeks old mice. Transgenic mice specifically deficiency CD147 in CD4^+^ T cells were generated by Shanghai Biomodel Organism Science and Technology Development Co. PCR screening was performed on transgenic offspring (CD4*^cre^*Bsg*^fl/fl^* and Bsg*^fl/fl^*) using the specified forward (CATGTCCATCAGGTTCTTGC) and reverse primers (CCAGGGTCGGAGACAATAAC). The Central South University provided specific pathogen-free conditions for housing all the mice. Approval for the animal study was granted by the Ethics Committee of Xiangya Hospital (the ethical approval permit numbers: 2019030227).

**IMQ-induced psoriasis-like dermatitis model**

Six- to eight-week-old mice were used for experiments. A daily dose of 31.25 mg of 5% IMQ cream was given to mice in one ear, and two ears were applied. No adjuvant was used during the model development. IMQ cream was treated for 6 days. Mice were sacrificed on Day7. The clinical skin scores were measured every other day until day 7 of treatment with IMQ according to the modified PASI (*4*). There were four degrees of skin lesions: 0 = no symptoms; 1 = mild; 2 = moderate; 3 = severe; and 4 = very severe. The thickness of mouse ear margin was measured with vernier caliper. Histological evaluation of mouse ears samples was performed by H&E staining. The thickness of the epidermis was measured from the stratum basale to the stratum granulosum using Image Pro-Plus (Image Pro-Plus 6.0 image-analysis software). The average value from seven random fields of view was calculated for each mouse.

**IL-23-induced psoriasis-like dermatitis mouse model**

The IL-23-induced psoriasis-like mouse model of psoriasis was establised as previously described (*5*). The ears of C57BL/6J mice were injected intradermally with 1 μg rmIL-23 (Sino Biological Cat. CT028-M08H) dissolved in 20 μl PBS into one ear and 20 μl PBS into the contralateral ear. Injections were used persistently every other day for a total of 8 days (days 1, 3, 5, and 7). On the eighth day, ears were analyzed by histopathology and flow analysis.

**IMQ-induced psoriasis-like recurrence model**

A daily dose of 62.5 mg of 5% IMQ cream was given to shaved back of mice, or a daily dose of 31.25 mg of 5% IMQ cream was given to mice in one ear and two ears were applied. No adjuvant was used during the model development. IMQ cream was treated for 6 days. And then all mice rested for 2 weeks. Mice were then re-challenged with IMQ for 6 days. Skin tissue were harvested on Day 7, Day 20 and Day 28, respectively. Skin tissue were analyzed by histopathology and flow analysis.

***In vitro* mouse T cell differentiation**

Mouse naïve CD4^+^ T cells were obtained from the spleen by using the CD4^+^CD62L^+^ T Cell Isolation Kit (Miltenyi Biotech Cat. 130-106-643). Mouse naïve CD4^+^ T cells were stimulated with anti-CD3 (plate-bound, 5 μg/ml, eBioscience Cat. 145-2c11) and anti-CD28 (2 μg/ml, BD Cat. 553294) in RPMI 1640 containing 10% FBS, 1000× 2-mercaptoethanol, 100× Sodium Pyruvate, 100× HEPES Buffer, 100× Non-Essential Amino Acids Solution, and 100× penicillin/streptomycin Nystatin Solution. For pathogenic Th17 cells polarization, naïve CD4^+^ T cells were activated with IL-6 (30 ng/ml, R&D Systems Cat. 406-ML-005), IL-1β (10 ng/ml, PeproTech Cat. 211-11B-10), IL-23 (20 ng/ml, R&D Systems Cat. 1887-ML-010), and TGF-β (2 ng/ml, R&D Systems Cat. 7666-MB-005) in the presence of anti-IFN-γ (10 μg/ml, BioXcell Cat. BE0054) and anti-IL-4 (10 μg/ml, BioXcell Cat. BE0045). For non-pathogenic Th17 cells differentiation, naïve CD4^+^ T cells were activated with IL-6 (30 ng/ml, R&D Systems Cat. 406-ML-005), IL-1β (10 ng/ml, PeproTech Cat. 211-11B-10), and TGF-β (2 ng/ml, R&D Systems Cat. 7666-MB-005) in the presence of anti-IFN-γ (10 μg/ml, BioXcell Cat. BE0054) and anti-IL-4 (10 μg/ml, BioXcell Cat. BE0045). The T cells were cultured for 3 days and were collected to detect Th17 differentiation by flow cytometry.

**Western blotting**

Radio immunoprecipitation assay buffer was used to lyse skin lesions and cells (Bimake Cat. B14002). 10 µg protein was run by SDS-PAGE and transferred to a PVDF membrane. After being blocked with 5% bovine serum albumin (BSA) for 1 hour, we incubated the membranes overnight with primary antibodies at 4 °C. The membranes were incubated with horseradish peroxidase-conjugated secondary antibodies for 1 hour at room temperature and then visualized by using a chemiluminescence substrate. Image Lab software (Bio-Rad) was used to analyze the images. Rabbit anti-p-JAK2 Ab (CST Cat. 3771), rabbit anti-p-STAT3 Ab (CST Cat. 9145), mouse anti-IL-6R Ab (Santa Cruz Cat. sc-373708), rabbit anti-p-SMAD2 Ab (CST Cat. 18338), rabbit anti-SMAD4 Ab (CST Cat. 46535), rabbit anti-RORγT Ab (Abcam Cat. ab78007), mouse anti-TGF-β R1 Ab (Santa Cruz Cat. sc-101574), mouse anti-TGF-β R2 Ab (Santa Cruz Cat. sc-17791), mouse anti-IL-15R (Santa Cruz Cat. sc-374023), or mouse anti-GAPDH Ab (Proteintech Cat. 6004-1-Ig) was used.

**Immunofluorescence staining and imaging**

Mouse CD4^+^ T cells were obtained from the spleen by using the CD4^+^ T Cell Isolation Kit (Miltenyi Biotech Cat. 130-117-043). The following primary antibodies were used: IL-6R mouse monoclonal IgG (Santa Cruz Biotechnology Cat. sc-373708), CD147 Polyclonal antibody (Proteintech Cat. 11989-1-AP), CD63 Rabbit mAb (Abcam Cat. ab217345), or mouse anti-IL-15R Ab (Santa Cruz Biotechnology Cat. sc-374023). CD4^+^ T cells were plated on 24-well chamber slides overnight and treated with IL-6 (R&D Systems Cat. 406-ML-005, 30 ng/mL) or IL-15 (R&D Systems Cat. 447-ML-010, 20 ng/mL) for 30 min. And then, CD4^+^ T cells were washed three times, fixed with 95% ethanol in D-PBS, and blocked with 5% BSA in D-PBS. Samples were then rinsed three times with D-PBS and incubated with secondary antibodies for 1 h at room temperature. Coverslips were mounted onto microscope slides with Fluoroshield Mounting Medium with DAPI (Abcam Cat. ab104139). Immunofluorescence and colocalization analysis were performed using confocal microscopy (Zeiss LSM 900 + Airyscan2 (Carl Zeiss)).

**Quantitative Real-Time PCR (RT-qPCR)**

TRIpure Reagent (Bioteke Cat. RP1001) was used to extract RNA from cells. HiScript II Q RT SuperMix for qPCR was used to convert RNA to cDNA (Vazyme Cat. R223-01). RT-qPCR was performed on a 7500 Fast thermocycler (Applied Biosystems) using the UltraSYBR Kit (CWBIO Cat. CW0659). The relative quantitative gene expression of target genes was measured by using the 2^–ΔΔCt^ method, and *β-Actin* was used as the endogenous control gene All primer sequences used for RT-qPCR were as follows: *Il-6r*: forward, 5′ - GCGTTTCACAGCTTAAAAATGG -3′, and reverse, 5′ - TGAACTCCTTTGACCATACAGG -3′; *Il-15r*: forward, 5′ - CCACAGTTCCAAAATGACGAAA -3′, and reverse, 5′ - GTACTGTTTCCATGGTTTCCAC -3′; *β-Actin*: forward, 5′ - AGAGCTACGAGCTGCCTGAC -3′, and reverse, 5′ - AGCACTGTGTTGGCGTACAG -3′.

**Protein extraction and Co-Immunoprecipitation (Co-IP)**

Mouse CD4^+^ T cells were obtained from the spleen by using the CD4^+^ T Cell Isolation Kit (Miltenyi Biotech Cat. 130-117-043). Whole-cell protein extracts were lysed in NP40 buffer, and protein concentrations were determined by a BCA Protein Assay Kit (Bioteke Corporation). For Co-IP, harvested cells were incubated in NP40 on ice for 30 min, and lysates were centrifuged at 14,000 rpm for 10 min to remove cellular debris. Precleared cell lysates were incubated with 20 μL of agarose beads with rotation for 1 h at 4 °C and were then centrifuged at 3000 rpm for 3 min to collect supernatants, into which 1.5 μg of specific antibodies were added and incubated for 1 h before adsorption to protein A/G agarose beads (Beyotime Biotechnology). After rotation overnight at 4 °C, all beads were recovered and washed three times with NP40. Then, 20 μL of lysis buffer and 10 μL of 5× loading buffer were added to the beads and incubated at 95 °C for 10 min. CD147 Polyclonal antibody (Proteintech Cat. 11989-1-AP), mouse anti-IL-6R Ab (Santa Cruz Cat. sc-373708), mouse anti-IL-15R Ab (Santa Cruz Cat. sc-374023), or Anti-Flag tag mouse monoclonal antibody (Sangon Biotech Cat. D191041) was used.

**RNA Sequencing and Transcriptomics Analysis**

We isolated total RNA and sequenced it at the Shenzhen Genomics Institute (Shenzhen, China) using a BGISEQ-500 platform. Bowtie2 aligned high-quality reads to the mouse reference genome (GRCm38). Expectation Maximization was used to normalize gene expression to fragments per kilobase per million mapped reads from RNA-Seq. Significant differential expression was set if a gene with > 2-fold expression difference versus the control with an adjusted p-value of < 0.05. AMIGO and DAVID software were used to analyze differentially expressed genes (DEGs). KEGG annotations were used to analyze DEG enrichment degrees. The accession number of RNA-seq dataset was PRJNA933282.

**Statistical analysis**

Statistical analysis was conducted using the software GraphPad Prism 8.0. The two groups were compared using Student's t test. An analysis of variance (ANOVA) was conducted, involving multiple comparisons. The Pearson Correlation Test was utilized for the purpose of examining correlation. The level of statistical significance was set to p < 0.05. *p < 0.05; **p < 0.01; ***p < 0.001; ****p < 0.0001. The data is expressed as means ± SEM.

Count (%) was used to express all data in the predictive nomogram, like demographic characteristics, the expression of CD147 on CD4^+^ T cells, and the expression of CD147 on Th17 cells (Table S3 and Table S5). The outcome measure in the efficacy predictive model is PASI75 responder or non-responders. The outcome measure in the recurrence predictive model is psoriasis patients with (or without) recurrence within 1 year after treatment. The R software (Version 3.6.3; https //www.R-project.org) was utilized for conducting statistical analysis. The optimal predictive features were selected using univariate logistic regression analysis (Table S4 and Table S6). The characteristics were evaluated in terms of odds ratio (OR) with a 95% confidence interval (CI) and p-value. All the levels of statistical significance were two-sided. A prediction model was developed using variables that had a p-value less than 0.05 in the analysis of logistic regression. The multivariable logistic regression analysis was used to build the prediction model. The formulas of the prediction model were shown in Figure legend S2. To assess the calibration of the predictive model, a calibration curve was plotted. To assess the discriminant performance of predictive model, the C-index was quantified. Moreover, the area under the curve (AUC) was graphed to test the predictive capability of the predictive model. The predictive model underwent validation through bootstrapping with 1,000 bootstrap resamples to determine a relatively adjusted C-index (*6*). To evaluate the clinical utility of the predictive model, decision curve analysis was conducted to measure the net rate of return across various threshold probabilities (*7*).

| **REAGENT or RESOURCE** | **SOURCE** | **IDENTIFIER** |
| --- | --- | --- |
| **Antibodies** | | |
| Anti-human CD4 – BV421 | BioLegend | Clone: RPA-T4; Cat#: 300532; RRID: AB_10965645 |
| Anti-human CD147 – AF488 | BioLegend | Clone: HIM6; Cat#: 306207; RRID: AB_528739 |
| Anti-human IL-17A – BV711 | BioLegend | Clone: BL168; Cat#: 512328; RRID: AB_2563888 |
| Anti-mouse CD45 – PerCP/Cyanine5.5 | BioLegend | Clone: 30-F11; Cat#: 103131; RRID: AB_893344 |
| Anti-mouse CD4 – BV421 | BioLegend | Clone: GK1.5; Cat#: 100443; RRID: AB_2562557 |
| Anti-mouse IFN-γ – AF488 | BioLegend | Clone: XMG1.2; Cat#: 505815; RRID: AB_493313 |
| Anti-mouse IL-17A – APC | BioLegend | Clone: TC11-18H10.1; Cat#: 506915; RRID: AB_536017 |
| Anti-mouse CD69 – APC/Cyanine7 | BioLegend | Clone: H1.2F3; Cat#: 104526; RRID: AB_10679041 |
| Anti-mouse CD103 – Alexa Fluor 700 | BioLegend | Clone: 2E7; Cat#: 121441; RRID: AB_2813992 |
| Phospho-Jak2 (Tyr1007/1008) Rabbit mAb (WB) | CST | Cat#: 3771 |
| Phospho-Stat3 (Tyr705) (D3A7) Rabbit mAb (WB) | CST | Cat#: 9145 |
| Phospho-SMAD2 (Ser465/Ser467) (E8F3R) Rabbit mAb (WB) | CST | Cat#: 18338 |
| SMAD4 (D3R4N) Rabbit mAb (WB) | CST | Cat#: 46535 |
| Anti-RORγT antibody (WB) | Abcam | Cat#: ab78007 |
| mouse anti-TGF-β R1 Ab (WB) | Santa Cruz Biotechnology | Cat#: sc-101574 |
| mouse anti-TGF-β R2 Ab (WB) | Santa Cruz Biotechnology | Cat#: sc-17791 |
| mouse anti-IL-15R Ab (WB, IF, IP) | Santa Cruz Biotechnology | Cat#: sc-374023 |
| mouse anti-IL-6R Ab (WB, IF, IP) | Santa Cruz Biotechnology | Cat#: sc-373708 |
| CD147 Polyclonal antibody (IF, IP) | Proteintech | Cat#: 11989-1-AP |
| CD63 Rabbit mAb (IF) | Abcam | Cat#: ab217345 |
| Anti-Flag tag mouse monoclonal antibody | Sangon Biotech | Cat#: D191041 |
| GAPDH Monoclonal Antibody (WB) | Proteintech | Cat#: 60004-1-Ig |
| CD3e Monoclonal Antibody | eBioscience | Cat#: MA5-17655 |
| Purified NA/LE Hamster Anti-Mouse CD28 | BD Biosciences | Cat#: 553294 |
| **Biological Samples** | | |
| Blood samples from healthy donors | Xiangya Hospital of Central South University | N/A |
| Blood samples from psoriasis patients | Xiangya Hospital of Central South University | N/A |
| **Chemicals, Peptides, and Recombinant Proteins** | | |
| Imiquimod Cream | Med-shine Pharmaceutical Co., Ltd | N/A |
| Ionomycin | TOCRIS | Cat#: 56092-8-0 |
| PMA | Sigma-Aldrich | Cat#: 79346 |
| GolgiPlug | BD Biosciences | Cat#: 555029 |
| Collagenase, Type IV | Sigma-Aldrich | Cat#: V900893 |
| Dispase II | Sigma-Aldrich | Cat#: D4693 |
| RmIL-23 | Sino Biological | CT028-M08H |
| Cell-Tak Cell and Tissue Adhesive | Corning | Cat#: 354240 |
| TRIpure Reagent | Bioteke | Cat#: RP1001 |
| HiScript II Q RT SuperMix for qPCR (+gDNA wiper) | Vazyme | Cat#: R223-01 |
| Recombinant Mouse IL-6 | R&D Systems | Cat#: 406-ML-005 |
| Recombinant Mouse TGF-β | R&D Systems | Cat#: 7666-MB-005 |
| Recombinant Mouse IL-23 | R&D Systems | Cat#: 1887-ML-010 |
| Recombinant Mouse IL-1β | PeproTech | Cat#: 211-11B-10 |
| InVivoMab anti-mouse IFN-γ | Bio X Cell | Cat#: BE0054 |
| InVivoMab anti-mouse IL-4 | Bio X Cell | Cat#: BE0045 |
| Recombinant Mouse IL-15 Protein | R&D Systems | Cat#: 447-ML-010 |
| **Critical Commercial Assays** | | |
|  | | |
| UltraSYBR One Step RT-qPCR Kit | CWBIO | Cat#: CW0659 |
| CD4^+^CD62L^+^ T Cell Isolation Kit, mouse | Miltenyi Biotech | Cat#: 130-106-643 |
| CD4^+^ T Cell Isolation Kit, mouse | Miltenyi Biotech | Cat#: 130-117-043 |
| Zombie Aqua™ Fixable Viability Kit | BioLegend | Cat#: 423102 |
| Foxp3/Transcription Factor Staining Buffer Kit | eBioscience | Cat#: 00-5523-00 |
| **Deposited Data** | | |
| Mouse Th17 RNA-seq | This paper | PRJNA933282 |
| **Experimental Models: Cell Lines** | | |
| Mouse naïve CD4^+^ Tcells | This paper | N/A |
| Mouse CD4^+^ Tcells | This paper | N/A |
| HEK293T | ATCC | CRL-3216 |
| **Experimental Models: Organisms/Strains** | | |
| Mouse: BALB/C | Hunan SJA Laboratory Animal Co., Ltd | N/A |
| Mouse: C57 | Hunan SJA Laboratory Animal Co., Ltd | N/A |
| **Oligonucleotides** | | |
| Mouse *Il6r* forward: GCGTTTCACAGCTTAAAAATGG | This paper | N/A |
| Mouse *Il6r* reverse: TGAACTCCTTTGACCATACAGG | This paper | N/A |
| Mouse *Il15r* forward: CCACAGTTCCAAAATGACGAAA | This paper | N/A |
| Mouse *Il15r* reverse: GTACTGTTTCCATGGTTTCCAC | This paper | N/A |
| Mouse *β-actin* forward: AGAGCTACGAGCTGCCTGAC | This paper | N/A |
| Mouse *β-actin* reverse: AGCACTGTGTTGGCGTACAG | This paper | N/A |
| **Software and Algorithms** | | |
| GraphPad Prism 8 | GraphPad | https://www.graphpad.com/ |
| FlowJo v10.4 | Tree Star | https://www.flowjo.com/ |
| Image Lab (Version 5.0) | Bio-Rad | https://www.bio-rad.com/ |
| Image Pro Plus (Version 6.0) | Media Cybernetics | https://partner.mediacy.com/ |
| R software (Version 3.6.3) |  | https://www.R-project.org |

**
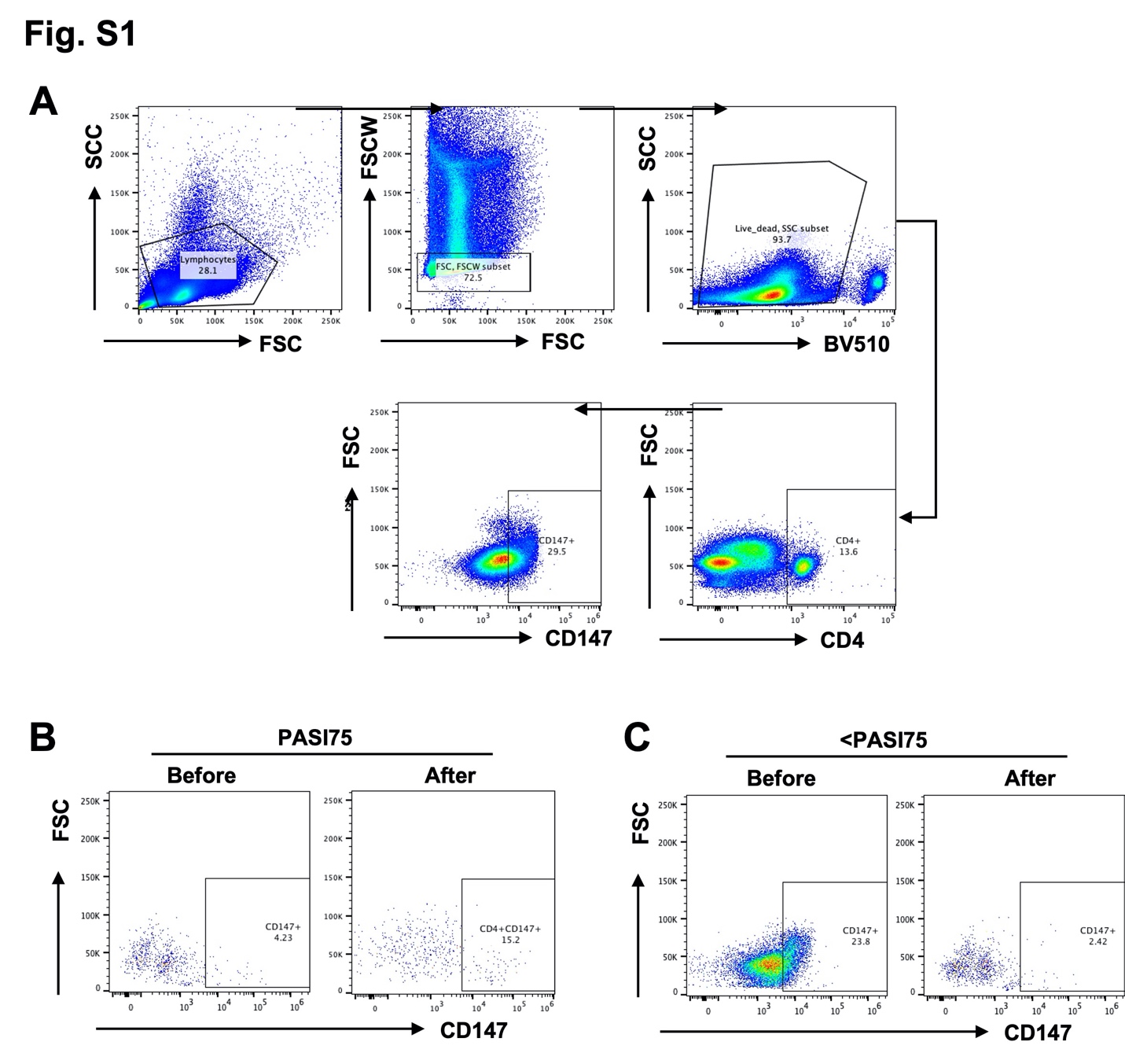
**

**Figure S1. CD147 expression on CD4^+^ T cells is highly correlated with the treatment efficacy and disease recurrence**

**(A)** All cells are gated with FSC/SSC for the single cells gate. FSC/FSCW is used to exclude doublets. Next, a viability dye (BV510) is used to exclude dead cells. Gating strategy to identify the expression of CD147 on CD4^+^ T cells in the peripheral blood monocytes cells on Fig. 1A and Fig. 1B. **(B)** Representative Flow cytometry plots of Fig. 1A. **(C)** Representative Flow cytometry plots of Fig. 1B.


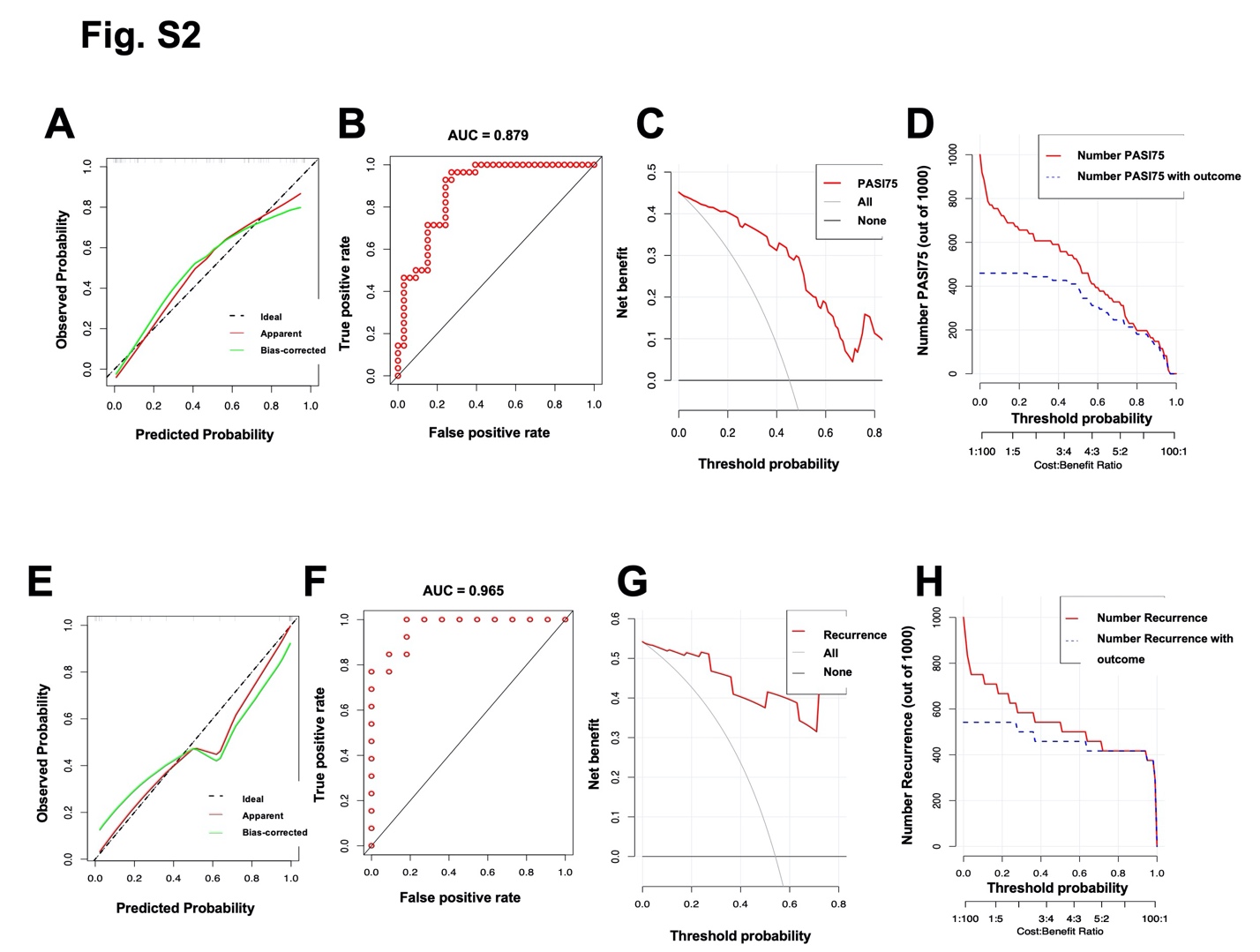


**Figure S2. The nomogram predicting the efficacy of IL-17A inhibitor and the risk of psoriasis recurrence based on CD147 expression on CD4^+^ T cells**

**(A-D)** The predictive nomogram of the PASI75 efficacy of IL-17A inhibitor for the treatment of psoriasis. We recruited the efficacy predictive model cohort (cohort 1). Sixty-four psoriasis patients were recruited and underwent a 4-week treatment with an IL-17A inhibitor. All patient data are shown in the Table S3. Thirty psoriasis patients achieved a PASI75 response, while 34 patients did not achieve a 75% response. Table S4 displays the outcomes of the univariate logistic regression analysis for the characteristics. The model included predictors that showed a notable difference (p < 0.05). These predictors consisted of sex, the expression of CD147 on CD4^+^ T cells (CD147_CD4^+^ T cells) before treatment, the expression of CD147 on Th17 cells (CD147_Th17 cells) before treatment, and BMI, which was based on the effect of the BMI on the effectiveness of IL-17A inhibitor (*8*) (Fig. 1G). The predictive equation was constructed as LogitP = 5.1157 + (-2.2166) * (Sex Male) + (-0.0795) * (BMI) + (-0.1305) * (CD147_CD4^+^ T cells) + (-0.0556) * (CD147_Th17 cells). The prediction model was suitable for predicting the probability of psoriasis patients achieving a PASI75 response following a 4-week treatment with an IL-17A inhibitor. An illustration is provided in Fig. 1G. For example, a female psoriasis patient with BMI 30 point. The expression of CD147 on CD4^+^ T cells was 10% and the expression of CD147 on Th17 cells was 20% in the PBMCs of psoriasis patient before the treatment of IL-17A inhibitor. According to the predictive equation, the possibility of this psoriasis patient achieving a PASI75 response was 57.10%. **(A)** The calibration curve of the nomogram showed good agreement. The C-index was 0.8855 (95% CI: 0.8012–0.9698) and was established as 0.8663 via bootstrapping validation. **(B)** The AUC of this prediction model was 0.879. **(C)** Decision curve for the efficacy predictive nomogram. **(D)** Clinical impact curve for the efficacy predictive nomogram. **(E-H)** The predictive nomogram of the risk of recurrence of psoriasis. In the recurrence predictive model cohort (cohort 2), 26 psoriasis patients were recruited and treated with an IL-17A inhibitor. Table S5 displays the attributes of the individuals. Fourteen psoriasis patients experienced recurrence within 1 year, while 12 patients did not experience recurrence. Table S6 displays the outcomes of the univariate logistic regression analysis for the characteristics. The predictors including BMI, the expression of CD147 on CD4^+^ T cells (CD147_CD4^+^ T cells) before treatment, the expression of CD147 on Th17 cells (CD147_Th17 cells) before treatment, the changes in the expression of CD147 on CD4^+^ T cells (CD147_CD4^+^ T cells (%)) after treatment with an IL-17A inhibitor for 4 weeks, and the changes in the expression of CD147 on Th17 cells (CD147_Th17 cells (%)) after 4 weeks treatment (Fig. 1H). The predictive equation was constructed as LogitP = -10.0101 + (0.4126) * (BMI) + (-0.0901) * (CD147_CD4+ T cells) + (0.1194) * (CD147_Th17 cells) + (-0.0031) * (CD147_CD4+ T cells (%)) + (-0.0115) * (CD147_Th17 cells (%)). The prediction model is suitable to predict the risk of recurrence of psoriasis within 1 year after treatment with an IL-17A inhibitor. Fig. 1H displays an illustration. For example, a psoriasis patient with BMI 20 point. The expression of CD147 on CD4^+^ T cells was 15% and the expression of CD147 on Th17 cells was 55% in the PBMCs of psoriasis patient before the treatment of IL-17A inhibitor. The changes in the expression of CD147 on CD4^+^ T cells was 600% and the changes in the expression of CD147 on Th17 cells was 100% in the PBMCs of psoriasis patient after the treatment of IL-17A inhibitor for 4 weeks. According to the predictive equation, the risk of recurrence of psoriasis within 1 year was 61.00%. **(E)** Calibration curves of recurrence predictive nomogram. The C-index was 0.965 (95% CI: 0.9046–1.0000) and was established as 0.8671 via bootstrapping validation. **(F)** The AUC of this prediction model was 0.965. **(G)** Decision curve for the recurrence predictive nomogram. **(H)** Clinical impact curve for the recurrence predictive nomogram.

**
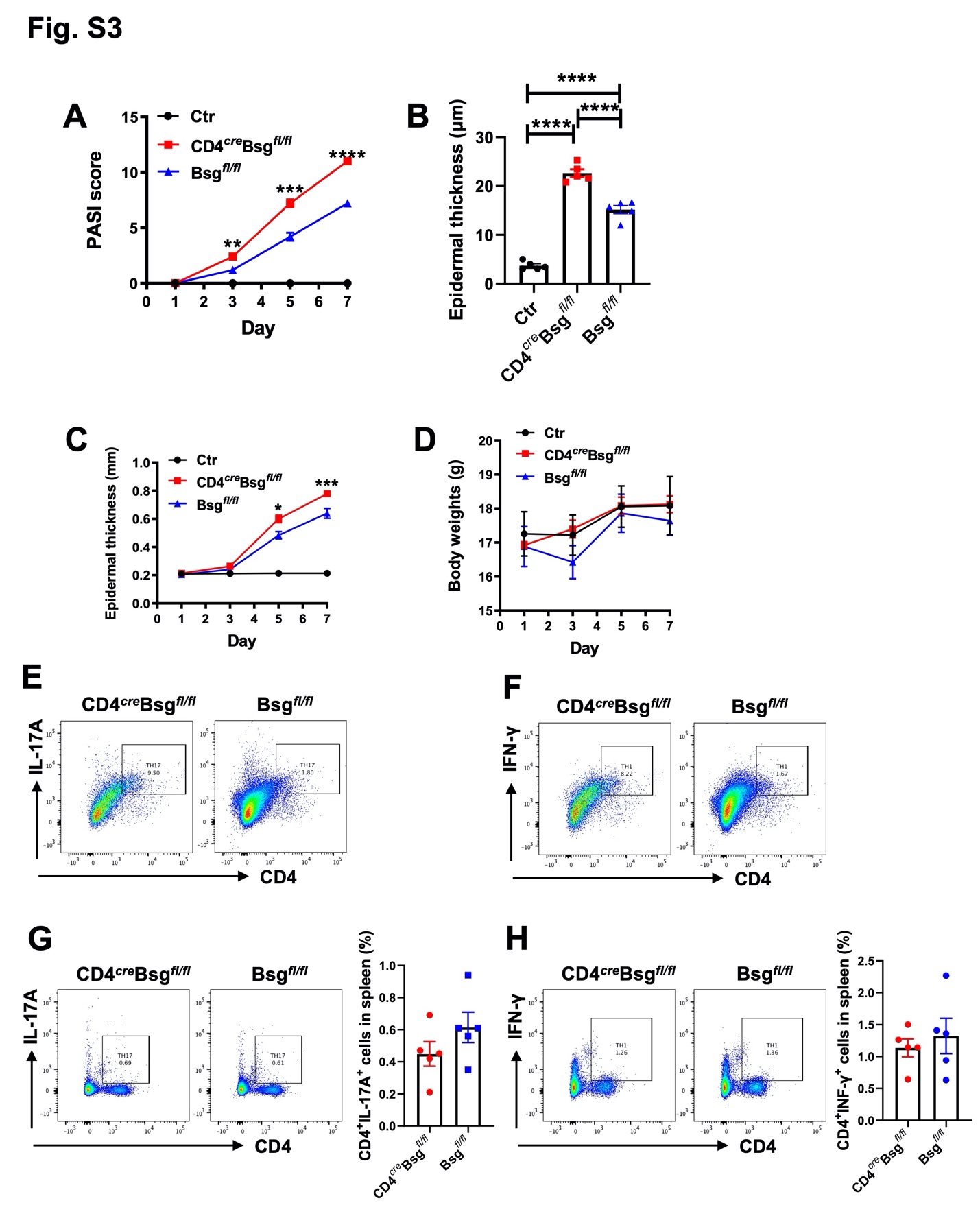
**

**Figure S3. Genomic knock out CD147 in CD4^+^ T cells aggravates IMQ-induced psoriasis-like dermatitis**

IMQ was applied for the CD4*^cre^*Bsg*^fl/fl^* and Bsg*^fl/fl^* mice ears. Five mice in each group were sacrificed on Day 7 to conduct experiments. **(A)** PASI scores. **(B)** Epidermal thickness. **(C)** Ear thickness measured by vernier caliper. **(D)** The body weights of mice. (**E-F**) Flow cytometric analysis of cell suspensions from the ear skin. **(E)** Representative Flow cytometry plots of Th17 (CD4^+^IL-17A^+^) cells. **(F)** Representative Flow cytometry plots of Th1 (CD4^+^IFN-γ^+^) cells. (**G-H**) Flow cytometric analysis of cell suspensions from the spleen. **(G)** Th17 (CD4^+^IL-17A^+^) cells. Representative Flow cytometry plots (left). Flow cytometric statistical data of Th17 (CD4^+^IL-17A^+^) cells in spleen from each group (n = 5) treated with IMQ for 6 days (right). **(H)** Th1 (CD4^+^IFN-γ^+^) cells. Representative Flow cytometry plots (left). Flow cytometric statistical data of Th1 (CD4^+^IFN-γ^+^) cells in spleen from each group (n = 5) treated with IMQ for 6 days (right). Data are presented as the mean ± SEM. *p < 0.05, **p < 0.01, ***p < 0.001, ****p < 0.0001.


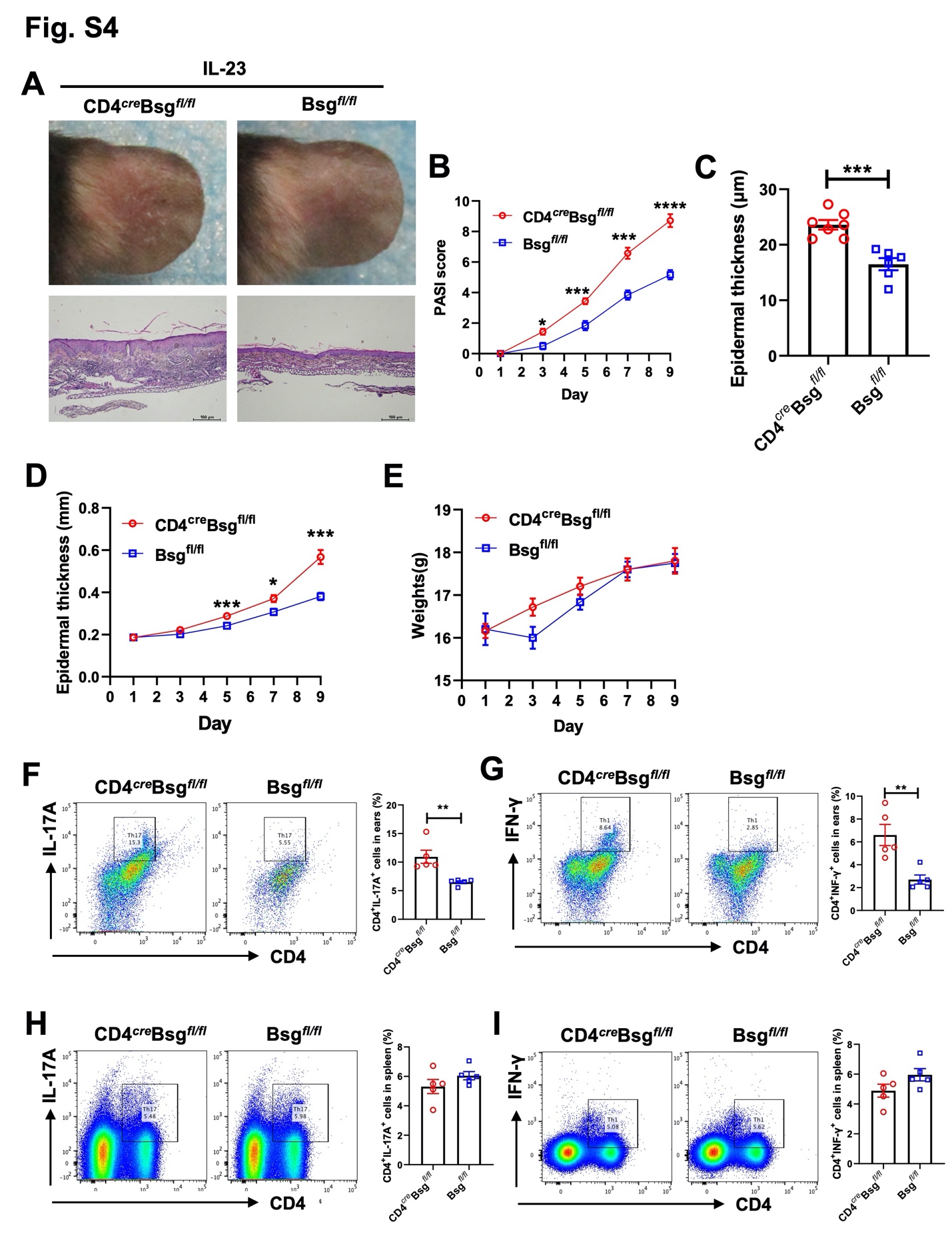


**Figure S4. Genomic knock out CD147 in CD4^+^ T cells aggravates IL-23-induced psoriasis-like dermatitis**

CD147 deficiency in CD4^+^ T cells aggravate the IL-23-induced psoriasis-like skin phenotype. IL-23 was injected into the CD4*^cre^*Bsg*^fl/fl^* and Bsg*^fl/fl^* mice ears (n = 5~7). **(A)** Phenotypic manifestation and H&E staining of CD4*^cre^*Bsg*^fl/fl^* and Bsg*^fl/fl^*. Scale bars, 100 μm. **(B)** PASI scores. **(C)** Epidermal thickness. **(D)** Ear thickness measured by vernier caliper. **(E)** The body weights of mice. **(F-G)** Flow cytometric analysis of cell suspensions from the ear skin. **(F)** Th17 (CD4^+^IL-17A^+^) cells. Representative Flow cytometry plots (left). Flow cytometric statistical data of Th17 (CD4^+^IL-17A^+^) cells in skin lesions from each group (n = 5) (right). **(G)** Th1 (CD4^+^IFN-γ^+^) cells. Representative Flow cytometry plots (left). Flow cytometric statistical data of Th1 (CD4^+^IFN-γ^+^) cells in skin lesions from each group (n = 5) (right). (**H-I**) Flow cytometric analysis of cell suspensions from the spleen. **(H)** Th17 (CD4^+^IL-17A^+^) cells. Representative Flow cytometry plots (left). Flow cytometric statistical data of Th17 (CD4^+^IL-17A^+^) cells in spleen from each group (n = 5) (right). **(I)** Th1 (CD4^+^IFN-γ^+^) cells. Representative Flow cytometry plots (left). Flow cytometric statistical data of Th1 (CD4^+^IFN-γ^+^) cells in spleen from each group (n = 5) (right). Data are presented as the mean ± SEM. *p < 0.05, **p < 0.01, ***p < 0.001, ****p < 0.0001.


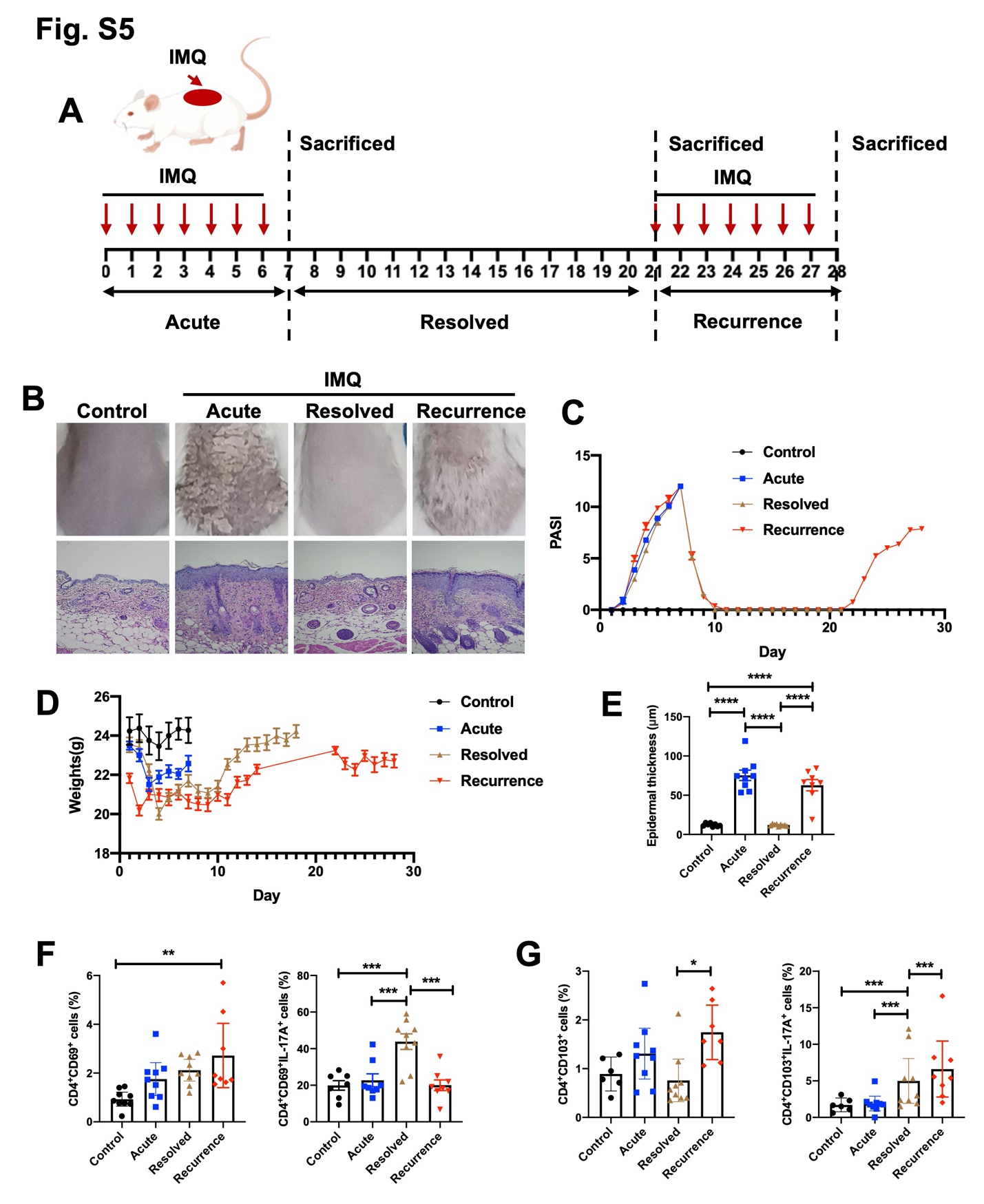


**Figure S5. IMQ-induced psoriasis-like recurrence mouse model**

**(A)** Schematic of the model. IMQ-induced psoriasis-like recurrence mouse model contains three stages: acute, resolved, and recurrence. **(B)** Phenotypic presentation and H&E staining of mice in Control, Acute, Resolved, and Recurrence group. (n = 7~9). **(C)** PASI scores of mice in **B** (n = 7~9). **(D)** The body weights of mice in **B** (n = 7~9). **(E)** Epidermal thickness was measured on HE-stained slides (n = 7~9). **(F-G)** Flow cytometric analysis of cell suspensions from the ear skin. **(F)** Flow cytometric statistical data of CD4^+^ CD69^+^ T_RM_ cells (left). Flow cytometric statistical data of the expression of IL-17A in CD4^+^ CD69^+^ T_RM_ cells (right) (n = 7~9). **(G)** Flow cytometric statistical data of CD4^+^ CD103^+^ T_RM_ cells (left). Flow cytometric statistical data of the expression of IL-17A in CD4^+^ CD103^+^ T_RM_ cells (right) (n = 7~9). Significant differences were evaluated using One-way ANOVA with Bonferroni’s post hoc test (**E-G**). Data are presented as the mean ± SEM. *p < 0.05, **p < 0.01, ***p < 0.001, ****p < 0.0001.


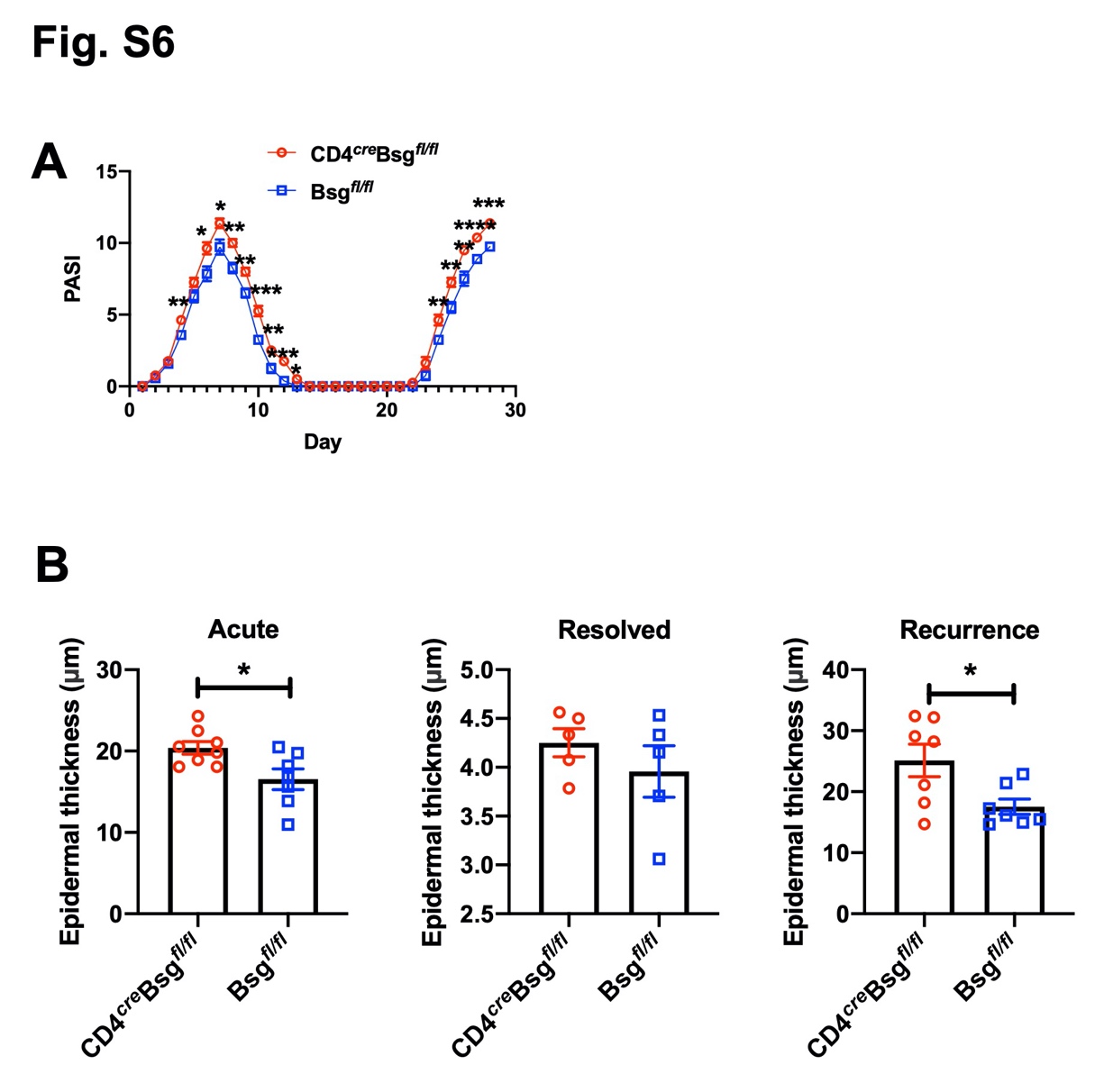


**Figure S6. CD147 deficiency in CD4^+^ T cells facilitates the recurrence of psoriasis**

IMQ was applied for the CD4*^cre^*Bsg*^fl/fl^* and Bsg*^fl/fl^* mice ears to established psoriasis-like recurrence mouse model as shown in Fig. S5. (n = 5~8). **(A)** PASI scores. **(B)** Epidermal thickness. Data are presented as the mean ± SEM. *p < 0.05.


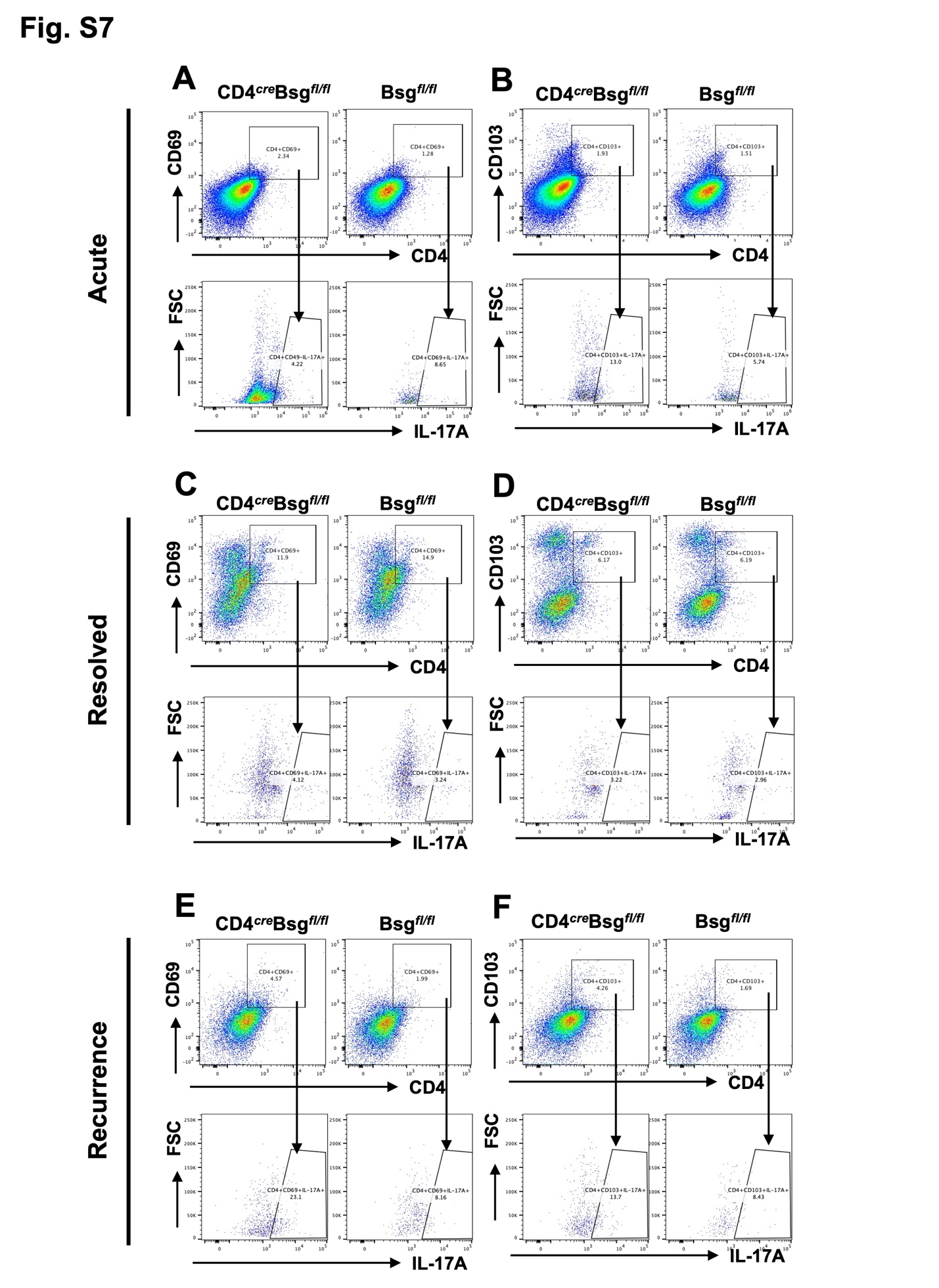


**Figure S7. CD147 deficiency in CD4^+^ T cells facilitates the infiltration of T_RM_ in skin lesions**

IMQ was applied for the CD4*^cre^*Bsg*^fl/fl^* and Bsg*^fl/fl^* mice ears to established psoriasis-like recurrence mouse model as shown in Fig. S5. (n = 5~8). **(A-F)** Flow cytometric analysis of cell suspensions from the ear skin. **(A-B)** Representative Flow cytometry plots of T_RM_ cells and the expression of IL-17A in T_RM_ cells in Acute group. **(A)** Representative Flow cytometry plots of CD4^+^ CD69^+^ T_RM_ cells and the expression of IL-17A in CD4^+^ CD69^+^ T_RM_ cells in Acute group. **(B)** Representative Flow cytometry plots of CD4^+^ CD103^+^ T_RM_ cells and the expression of IL-17A in CD4^+^ CD103^+^ T_RM_ cells in Acute group. **(C-D)** Representative Flow cytometry plots of T_RM_ cells and the expression of IL-17A in T_RM_ cells in Resolved group. **(C)** Representative Flow cytometry plots of CD4^+^ CD69^+^ T_RM_ cells and the expression of IL-17A in CD4^+^ CD69^+^ T_RM_ cells in Resolved group. **(D)** Representative Flow cytometry plots of CD4^+^ CD103^+^ T_RM_ cells and the expression of IL-17A in CD4^+^ CD103^+^ T_RM_ cells in Resolved group. **(E-F)** Representative Flow cytometry plots of T_RM_ cells and the expression of IL-17A in T_RM_ cells in Recurrence group. **(E)** Representative Flow cytometry plots of CD4^+^ CD69^+^ T_RM_ cells and the expression of IL-17A in CD4^+^ CD69^+^ T_RM_ cells in Recurrence group. **(F)** Representative Flow cytometry plots of CD4^+^ CD103^+^ T_RM_ cells and the expression of IL-17A in CD4^+^ CD103^+^ T_RM_ cells in Recurrence group.

**
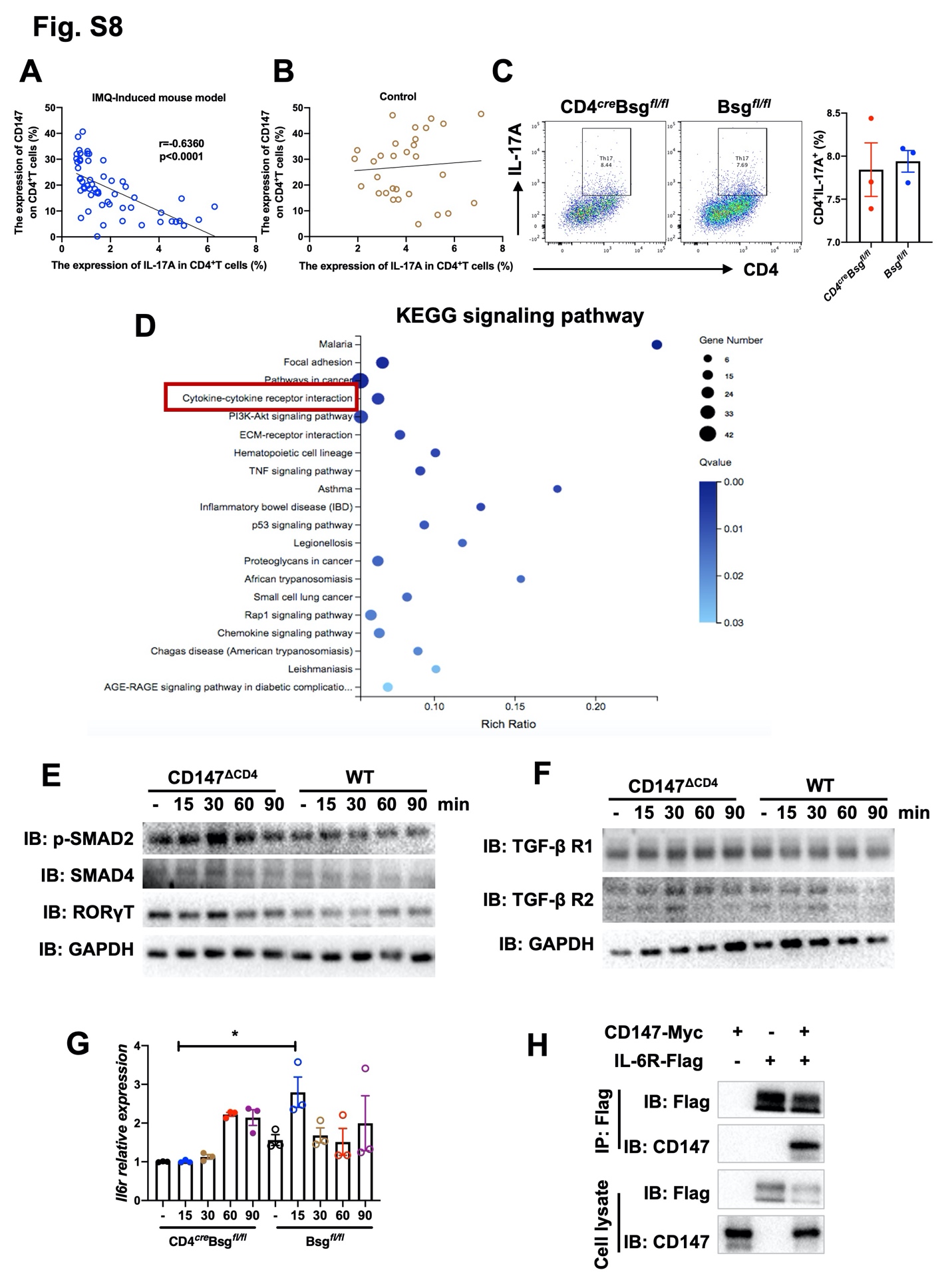
**

**Figure S8. CD147 expression in CD4^+^ T cells attenuates Th17 skewing through the endocytosis of IL-6R**

**(A)** The correlation analysis of the expression of CD147 and IL-17A in CD4^+^ T cells in spleen of psoriasis like mouse model. IMQ was applied for the mice (BALB/c) ears for 1 day (n = 26) and 3 days (n = 30) to established psoriasis-like mouse model. **(B)** The correlation analysis of the expression of CD147 and IL-17A in CD4^+^ T cells in spleen of control mice. (n = 30). **(C)** The effect of CD147 on the differentiation of non‐pathogenic Th17 cells. Mouse naïve CD4^+^ T cells were isolated from the spleen of CD4*^cre^*Bsg*^fl/fl^* and Bsg*^fl/fl^* mice and activated *in vitro* for 3 days with CD3/CD28 antibodies under non‐pathogenic Th17-polarizing conditions. The percentage of CD4^+^IL-17A^+^ non‐pathogenic Th17 cells was detected by flow cytometry. Representative Flow cytometry plots (left). Flow cytometric statistical data of non‐pathogenic Th17 (CD4^+^IL-17A^+^) cells (n = 3). **(D)** KEGG pathway enrichment in CD4^+^ T cells of CD4*^cre^*Bsg*^fl/fl^* and Bsg*^fl/fl^* mice under Th17-polarizing conditions. Mouse naïve CD4^+^ T cells were isolated from the spleen of CD4*^cre^*Bsg*^fl/fl^* and Bsg*^fl/fl^* mice and activated *in vitro* for 3 days with CD3/CD28 antibodies under Th17-polarizing conditions. Total RNA was extracted from cells and then performed by RNA sequencing. p value cutoff = 0.01. **(E)** CD147 inhibits the activation of p-SMAD2-RORγT signaling pathway in CD4^+^ T cells. Mouse CD4^+^ T cells were isolated from the spleen of CD4*^cre^*Bsg*^fl/fl^* and Bsg*^fl/fl^* mice and activated *in vitro* with TGF-β (2 ng/mL) for 15, 30, 60, or 90 min. Cells were extracted and subjected to immunoblot analysis using antibodies to p-SMAD2, SMAD4, or RORγT. GAPDH was used as a control. **(F)** The effect of CD147 on the expression of TGF-β R1 or TGF-β R2. CD4^+^ T cells isolated from the spleen of CD4*^cre^*Bsg*^fl/fl^* and Bsg*^fl/fl^* mice and activated *in vitro* with TGF-β (2 ng/mL) for 15, 30, 60, or 90 min. Cells were extracted and subjected to immunoblot analysis using antibodies to TGF-β R1 or TGF-β R2. GAPDH was used as a control. **(G)** The effect of CD147 on the mRNA expression of *Il6r* in CD4^+^ T cells. CD4^+^ T cells obtained from the spleen of CD4*^cre^*Bsg*^fl/fl^* and Bsg*^fl/fl^* mice and activated *in vitro* with IL-6 (30 ng/mL) for 15, 30, 60, or 90 min. **(H)** CD147 interacts with IL-6R on 293T cells. 293T cells were over-expressed by CD147-Myc, IL-16R-Flag, or CD147-Myc + IL-16R-Flag. Cells extracts were used for immunoprecipitation with a Flag antibody. The immunoprecipitated complex was detected by western blotting with the indicated antibodies.

**Supplementary Table 1. Demographics and clinical characteristics of the psoriasis patients.**

| **Characteristics** | **Psoriasis patients**  **(n =64)** |
| --- | --- |
| **Sex** |  |
| Male | 49 |
| Female | 15 |
| **Age, years, mean ± SEM (range)** | 38.22 ± 12.12 (9-64) |
| **Age at onset of psoriasis, years, mean ± SEM (range)** | 27.91 ± 12.86 (3-59) |
| **Duration of psoriasis, months, mean ± SEM (range)** | 125.2 ± 98.21 (3-432) |
| **Disease process** |  |
| Progressive phase | 55 |
| Stable phase | 8 |
| Regressive phase | 1 |
| **Family history ^1^** |  |
| No | 52 |
| Yes | 12 |
| **Smoking** |  |
| No | 35 |
| Yes | 29 |
| **Drinking** |  |
| No | 40 |
| Yes | 24 |
| **BMI ^2^, mean ± SEM** | 24.28 ± 4.111 |
| **PASI (before treatment) ^3^, mean ± SEM** | 13.33 ± 8.059 |
| **DLQI ^4^, mean ± SEM** | 7.344 ± 4.748 |

1: Family history, family history of plaque psoriasis.

2: BMI, body mass index

3: PASI, psoriasis area and severity index.

4: DLQI, dermatology life quality index.

**Supplementary Table 2. Demographics and clinical characteristics of the psoriasis patients treated with IL-17A inhibitor.**

| **Characteristics** | **Psoriasis patients**  **(n =26)** |
| --- | --- |
| **Sex** |  |
| Male | 23 |
| Female | 3 |
| **Age, years, mean ± SEM (range)** | 40.69 ± 9.536 (24-56) |
| **Age at onset of psoriasis, years, mean ± SEM (range)** | 29.08 ± 11.06 (5-50) |
| **Duration of psoriasis, months, mean ± SEM (range)** | 134.9 ± 88.05 (8-288) |
| **Disease process** |  |
| Progressive phase | 23 |
| Stable phase | 2 |
| Regressive phase | 1 |
| **Family history ^1^** |  |
| No | 19 |
| Yes | 7 |
| **Smoking** |  |
| No | 12 |
| Yes | 14 |
| **Drinking** |  |
| No | 16 |
| Yes | 10 |
| **BMI ^2^, mean ± SEM** | 24.50 ± 3.454 |
| **PASI (before treatment) ^3^, mean ± SEM** | 14.95 ± 7.263 |
| **PASI% ^4^, mean ± SEM** | 57.29 ± 38.00 |
| **DLQI ^5^, mean ± SEM** | 7.615 ± 4.570 |
| **Recurrence ^6^** |  |
| No | 12 |
| Yes | 14 |

1: Family history, family history of plaque psoriasis.

2: BMI, body mass index

3: PASI, psoriasis area and severity index.

4: PASI%, The improvement of PASI score (PASI%) = (the PASI score of psoriasis patients before the treatment of biologic therapy - the PASI score of psoriasis patients after the treatment of biologic therapy for 4 weeks) / the PASI score of psoriasis patients before the treatment of biologic therapy.

5: DLQI, dermatology life quality index.

6: Recurrence, psoriasis patients did / did not recurrence within 1 year of standardized treatment with biological agents

**Supplementary Table 3. Differences between demographic and clinical characteristics of psoriasis patients with or without PASI75.**

| **Characteristics** | **Psoriasis patients treated with IL-17A inhibitor (%)** | | |
| --- | --- | --- | --- |
|  | **PASI75**  **(n = 30)** | **<PASI75**  **(n = 34)** | **Total (n = 64)** |
| **Sex** |  |  |  |
| Male | 18 (60.00%) | 31 (91.18%) | 49 (76.56%) |
| Female | 12 (40.00%) | 3 (8.82%) | 15 (23.44%) |
| **Age (years)** |  |  |  |
| <40 | 18 (60.00%) | 19 (55.89%) | 37 (57.81%) |
| ≥40 | 12 (40.00%) | 15 (44.11%) | 27 (42.19%) |
| **Age at onset (years)** |  |  |  |
| <40 | 24 (80.00%) | 26 (76.47%) | 50 (78.13%) |
| ≥40 | 6 (20.00%) | 8 (23.53%) | 14 (21.88%) |
| **Duration (months)** |  |  |  |
| <60 | 14 (46.67%) | 9 (26.47%) | 23 (35.94%) |
| ≥60, <120 | 2 (23.53%) | 8 (23.53%) | 10 (15.63%) |
| ≥120, <180 | 5 (26.47%) | 9 (26.47%) | 14 (21.88%) |
| ≥180 | 9 (23.53%) | 8 (23.53%) | 17 (26.56%) |
| **Education** |  |  |  |
| Primary school or incomplete | 5 (16.67%) | 3 (8.82%) | 8 (12.50%) |
| Junior middle school graduate | 5 (16.67%) | 8 (23.53%) | 13 (20.31%) |
| High school graduate | 6 (20.00%) | 5 (14.71%) | 11 (17.19%) |
| Technical secondary school | 1 (3.33%) | 1 (2.94%) | 2 (3.13%) |
| University/college | 13 (43.33%) | 17 (50.00%) | 30 (46.88%) |
| **Cause** |  |  |  |
| No | 17 (56.67%) | 21 (61.77%) | 38 (59.38%) |
| Yes | 13 (43.33%) | 13 (38.24%) | 26 (40.63%) |
| **Disease process** |  |  |  |
| Progressive phase | 26 (86.67%) | 30 (88.24%) | 56 (81.25%) |
| Stable phase | 3 (10.00%) | 4 (11.76%) | 7 (10.94) |
| Regressive phase | 1 (3.33%) | 0 (0.00%) | 1 (1.56%) |
| **Family history ^1^** |  |  |  |
| No | 22 (73.33%) | 30 (88.24%) | 52 (81.25%) |
| Yes | 8 (26.67%) | 4 (11.76%) | 12 (18.75%) |
| **Smoking** |  |  |  |
| None | 19 (63.33%) | 16 (47.06%) | 35 (54.69%) |
| Not now | 1 (3.33%) | 6 (17.65%) | 7 (10.94%) |
| Now | 10 (33.34%) | 12 (35.29%) | 22 (34.38%) |
| **Drinking** |  |  |  |
| None | 21 (70.00%) | 19 (55.89%) | 33 (51.56%) |
| Not drinking for 1 year | 2 (6.67%) | 11 (32.35%) | 16 (25.00%) |
| ≤Once per week | 7 (23.33%) | 3 (8.82%) | 8 (12.50%) |
| >Once per week | 0 (0.00%) | 1 (2.94%) | 7 (10.94%) |
| **WHR ^2^** |  |  |  |
| Male ≤0.9 | 13 (43.33%) | 20 (58.82%) | 33 (51.56%) |
| >0.9 | 5 (16.67%) | 11 (32.35%) | 16 (25.00%) |
| Female ≤0.85 | 6 (20.00%) | 2 (5.88%) | 8 (12.50%) |
| >0.85 | 6(20.00%) | 1 (2.94%) | 7 (10.94%) |
| **BMI ^3^** |  |  |  |
| <18.5 | 2 (6.67%) | 3 (8.82%) | 5 (7.81%) |
| ≥18.5, <23.9 | 14 (46.67%) | 12 (35.29%) | 26 (40.63%) |
| ≥23.9, <28.0 | 12 (40.00%) | 10 (29.41%) | 22 (34.38%) |
| ≥28.0, <30.0 | 1 (3.33%) | 4 (11.77%) | 5 (7.81%) |
| ≥30.0 | 1 (3.33%) | 5 (14.71%) | 6 (9.38%) |
| **The type of plaque ^4^** |  |  |  |
| Small | 10 (3.33%) | 14 (41.18%) | 24 (37.50%) |
| Large | 20 (6.67%) | 20 (58.82%) | 40 (62.50%) |
| **PASI (before treatment) ^5^, mean ± SEM** | 14.19 ± 7.843 | 12.57 ± 8.287 | 13.33 ± 8.059 |
| **DLQI ^6^** |  |  |  |
| <10 | 21 (33.33%) | 24 (41.18%) | 24 (37.50%) |
| ≥10 | 9 (66.67%) | 10 (58.82%) | 40 (62.50%) |
| **The expression of CD147 on CD4^+^ T cells (CD147_CD4^+^ T cells) (%)** | 3.519 ± 3.189 | 14.28 ± 11.84 | 9.236 ± 10.36 |
| **The expression of CD147 on Th17 cells (CD147_Th17 cells) (%)** | 7.525 ± 9.363 | 29.00 ± 22.72 | 18.93 ± 20.67 |

1: Family history, family history of plaque psoriasis.

2: WHR, waist-to-hip ratio; male >0.9 defined as obese; female >0.85 defined as obese (*9, 10*).

3: BMI, body mass index (*10*).

4: The type of plaque, Small: psoriasis plaques are usually < 2 cm in diameter; Large: psoriasis plaques are usually ≥ 2 cm in diameter (*11*).

5: PASI, psoriasis area and severity index.

6: DLQI, dermatology life quality index.

**Supplementary Table 4. The univariate logistic regression analysis of psoriatic patients in cohort 1.**

| **Characteristics** | **Univariate analysis** | | |
| --- | --- | --- | --- |
|  | **β** | **Odds ratio (95% CI)** | **p-Value** |
| **Sex** | -1.930 | 0.145 (0.030-0.528) | 0.007 |
| **Age** | 0.100 | 0.905 (0.328-2.477) | 0.845 |
| **Age at onset** | -0.208 | 0.813 (0.236-2.676) | 0.733 |
| **Duration** | 0.442 | 1.556 (0.682-3.734) | 0.301 |
| **Education** | 0.511 | 1.667 (0.409-8.126) | 0.484 |
| **Cause** | 0.211 | 1.235 (0.453-3.387) | 0.679 |
| **Family history ^1^** | 1.003 | 2.727 (0.759-11.300) | 0.136 |
| **Smoking** | 0.172 | 1.188 (0.611-2.338) | 0.613 |
| **Drinking** | 0.100 | 1.105 (0.593-2.073) | 0.752 |
| **WHR ^2^** | -0.087 | 0.917 (0.398-2.091) | 0.835 |
| **BMI ^3^** | -0.008 | 0.992 (0.922-1.067) | 0.827 |
| **PASI (before treatment) ^4^** | 0.025 | 1.025 (0.964-1.094) | 0.421 |
| **DLQI ^5^** | 0.028 | 1.029 (0.346-3.03) | 0.959 |
| **The expression of CD147 on CD4^+^ T cells (CD147_CD4^+^ T cells) (%)** | -0.208 | 0.812 (0.701-0.902) | 0.001 |
| **The expression of CD147 on Th17 cells (CD147_Th17 cells) (%)** | -0.077 | 0.926 (0.879-0.962) | <0.001 |

**Supplementary Table 5. Differences between demographic and clinical characteristics of psoriasis patients with or without recurrence.**

| **Characteristics** | **Psoriasis patients treated with IL-17A inhibitor (%)** | | |
| --- | --- | --- | --- |
|  | **Recurrence**  **(n = 14)** | **No recurrence**  **(n = 12)** | **Total**  **(n = 26)** |
| **Sex** |  |  |  |
| Male | 13 (92.86%) | 10 (83.33%) | 23 (88.46%) |
| Female | 1 (7.14%) | 2 (16.67%) | 3 (11.54%) |
| **Age (years)** |  |  |  |
| <40 | 5 (35.71%) | 7 (58.33%) | 12 (46.15%) |
| ≥40 | 9 (64.29%) | 5 (41.67%) | 14 (53.85%) |
| **Age at onset (years)** |  |  |  |
| <40 | 10 (71.43%) | 11 (91.67%) | 21 (80.77%) |
| ≥40 | 4 (28.57%) | 1 (8.33%) | 5 (19.23%) |
| **Duration (months)** |  |  |  |
| <60 | 3 (21.43%) | 4 (33.33%) | 7 (26.92%) |
| ≥60, <120 | 3 (21.43%) | 2 (16.67%) | 5 (19.23%) |
| ≥120, <180 | 3 (21.43%) | 2 (16.67%) | 5 (19.23%) |
| ≥180 | 5 (35.71%) | 4 (33.33%) | 9 (34.62%) |
| **Education** |  |  |  |
| Primary school or incomplete | 1 (7.14%) | 1 (8.33%) | 2 (7.69%) |
| Junior middle school graduate | 5 (35.71%) | 1 (8.33%) | 6 (23.08%) |
| High school graduate | 3 (21.43%) | 1 (8.33%) | 4 (15.38%) |
| Technical secondary school | 1 (7.14%) | 0 (0.00%) | 1 (3.85%) |
| University/college | 4 (28.57%) | 9 (75.00%) | 13 (50.00%) |
| **Cause** |  |  |  |
| No | 8 (57.14%) | 8 (66.67%) | 16 (61.54%) |
| Yes | 6 (42.86%) | 4 (33.33%) | 10 (38.46%) |
| **Family history ^1^** |  |  |  |
| No | 10 (71.43%) | 9 (75.00%) | 19 73.08%) |
| Yes | 4 (28.57%) | 3 (25.00%) | 7 (26.92%) |
| **Smoking** |  |  |  |
| None | 4 (28.57%) | 8 (66.67%) | 12 (46.15%) |
| Not now | 2 (14.29%) | 1 (8.33%) | 3 (11.54%) |
| Now | 8 (57.14%) | 3 (25.00%) | 11 (42.31%) |
| **Drinking** |  |  |  |
| None | 9 (64.29%) | 7 (58.33%) | 16 (61.54%) |
| Not drinking for 1 year | 4 (28.57%) | 2 (16.67%) | 6 (23.08%) |
| ≤Once per week | 1 (7.14%) | 3 (25.00%) | 4 (15.38%) |
| >Once per week | 0 (0.00%) | 0 (0.00%) | 0 (0.00%) |
| **WHR ^2^** |  |  |  |
| Male ≤0.9 | 4 (28.57%) | 3 (25.00%) | 7 (26.92%) |
| >0.9 | 9 (64.29%) | 7 (58.33%) | 16 (61.54%) |
| Female ≤0.85 | 0 (0.00%) | 2 (16.67%) | 2 (7.69%) |
| >0.85 | 1(7.14%) | 0 (0.00%) | 1 (3.85%) |
| **BMI ^3^, mean ± SEM** | 24.97 ± 4.128 | 17.33± 10.68 | 21.47± 8.578 |
| **The type of plaque ^4^** |  |  |  |
| Small | 4 (28.57%) | 4 (33.33%) | 8 (30.77%) |
| Large | 10 (71.43%) | 8 (66.67%) | 18 (69.23%) |
| **PASI (before treatment) ^5^, mean ± SEM** | 14.20 ± 7.701 | 15.83 ± 6.945 | 14.95 ± 7.263 |
| **The improvement of PASI score (%) ^6^** | 24.97 ± 4.128 | 67.97 ± 42.04 | 57.29 ± 38.00 |
| **DLQI ^7^** |  |  |  |
| <10 | 10 (71.43%) | 8 (66.67%) | 18 (69.23%) |
| ≥10 | 4 (28.57%) | 4 (33.33%) | 8 (30.77%) |
| **The expression of CD147 on CD4^+^ T cells (before treatment) (CD147_CD4^+^ T cells) (%)** | 15.74 ± 9.871 | 7.56 ± 8.053 | 11.96 ± 9.823 |
| **The number of Th17 cells (before treatment) (%)** | 15.72 ± 15.66 | 14.59 ± 14.77 | 15.20 ± 14.96 |
| **The expression of CD147 on Th17 cells (before treatment) (CD147_Th17 cells) (%)** | 40.44 ± 18.13 | 21.97 ± 24.29 | 31.92 ± 22.77 |
| **The expression of CD147 on CD4^+^ T cells (after treatment) (CD147_CD4^+^ T cells) (%)** | 9.149 ± 9.465 | 10.09 ± 8.758 | 9.583 ± 8.975 |
| **The number of Th17 cells (after treatment) (%)** | 19.76 ± 15.13 | 17.10 ± 12.05 | 18.53 ± 13.60 |
| **The expression of CD147 on Th17 cells (after treatment) (CD147_Th17 cells) (%)** | 20.40 ± 20.21 | 27.81 ± 22.92 | 23.82 ± 21.39 |
| **The change of CD147 on CD4^+^ T cells (after treatment) (CD147_CD4^+^ T cells (%)) (%) ^8^** | -6.958 ± 94.06 | 374.3 ± 523.0 | 169.1 ± 403.3 |
| **The change of CD147 on Th17 cells (after treatment) (CD147_Th17 cells (%)) (%) ^9^** | -37.32 ± 62.63 | 156.8 ± 278.6 | 52.26 ± 214.3 |

1: Family history, family history of plaque psoriasis.

2: WHR, waist-to-hip ratio; male >0.9 defined as obese; female >0.85 defined as obese (*9, 10*).

3: BMI, body mass index (*10*).

4: The type of plaque, Small: psoriasis plaques are usually < 2 cm in diameter; Large: psoriasis plaques are usually ≥ 2 cm in diameter (*11*).

5: PASI, psoriasis area and severity index.

6: The improvement of PASI score (PASI%) = (the PASI score of psoriasis patients before the treatment of IL-17A inhibitor - the PASI score of psoriasis patients after the treatment of IL-17A inhibitor for 4 weeks)/the PASI score of psoriasis patients before the treatment of IL-17A inhibitor.

7: DLQI, dermatology life quality index.

8: The change of CD147 on CD4^+^ T cells (after treatment) (CD147_CD4^+^ T cells (%)) = (the expression of CD147 on the CD4^+^ T cells of psoriasis patients after the treatment of IL-17A inhibitor for 4 weeks - the expression of CD147 on the CD4^+^ T cells of psoriasis patients before the treatment of IL-17A inhibitor)/the expression of CD147 on the CD4^+^ T cells of psoriasis patients before the treatment of IL-17A inhibitor.

9: The change of CD147 on Th17 cells (after treatment) (CD147_Th17 cells (%)) = (the expression of CD147 on the Th17 cells of psoriasis patients after the treatment of IL-17A inhibitor for 4 weeks- the expression of CD147 on the Th17 cells of psoriasis patients before the treatment of IL-17A inhibitor)/the expression of CD147 on the Th17 cells of psoriasis patients before the treatment of IL-17A inhibitor.

**Supplementary Table 6. The univariate logistic regression analysis of psoriatic patients in cohort 2.**

| **Characteristics** | **Univariate analysis** | | |
| --- | --- | --- | --- |
|  | **β** | **Odds ratio (95% CI)** | **p-Value** |
| **Sex** | 0.955 | 2.600 (0.218-60.776) | 0.461 |
| **Age** | 0.070 | 1.073 (0.983-1.185) | 0.134 |
| **Age at onset** | 1.482 | 4.400 (0.536-94.102) | 0.217 |
| **Duration** | -0.288 | 0.750 (0.148-3.402) | 1.000 |
| **Education** | -2.066 | 1.000 (0.040-25.288) | 0.484 |
| **Cause** | 0.405 | 1.500 (0.305-7.910) | 0.619 |
| **Family history ^1^** | 0.182 | 1.200 (0.207-7.534) | 0.838 |
| **Smoking** | -0.693 | 0.500 (0.133-1.587) | 0.258 |
| **Drinking** | 0.251 | 1.286 (0.479-3.597) | 0.618 |
| **WHR ^2^** | 0.580 | 1.786 (0.350-9.699) | 0.486 |
| **BMI ^3^** | 0.172 | 1.188 (1.026-1.563) | 0.100 |
| **The type of plaque ^4^** | 0.223 | 1.250 (0.228-6.906) | 0.793 |
| **PASI (before treatment) ^5^** | -0.032 | 0.968 (0.862-1.080) | 0.562 |
| **The improvement of PASI score (%) ^6^** | -0.015 | 0.985 (0.960-1.006) | 0.189 |
| **DLQI ^7^** | -0.223 | 1.029 (0.346-3.03) | 0.793 |
| **The expression of CD147 on CD4^+^ T cells (before treatment) (CD147_CD4^+^ T cells) (%)** | 0.107 | 1.113 (1.013-1.262) | 0.048 |
| **The number of Th17 cells (before treatment) (%)** | 0.005 | 1.005 (0.953-1.065) | 0.846 |
| **The expression of CD147 on Th17 cells (before treatment) (CD147_Th17 cells) (%)** | 0.041 | 1.042 (1.004-1.090) | 0.046 |
| **The expression of CD147 on CD4^+^ T cells (after treatment) (CD147_CD4^+^ T cells) (%)** | -0.012 | 0.988 (0.900-1.082) | 0.786 |
| **The number of Th17 cells (after treatment) (%)** | 0.015 | 1.015 (0.958-1.081) | 0.614 |
| **The expression of CD147 on Th17 cells (after treatment) (CD147_Th17 cells) (%)** | -0.017 | 0.983 (0.944-1.020) | 0.375 |
| **The change of CD147 on CD4^+^ T cells (after treatment) (CD147_CD4^+^ T cells (%)) (%) ^8^** | -0.006 | 0.994 (0.986-0.998) | 0.046 |
| **The change of CD147 on Th17 cells (after treatment) (CD147_Th17 cells (%)) (%) ^9^** | -0.009 | 0.991 (0.978-0.998) | 0.048 |

1: Family history, family history of plaque psoriasis.

2: WHR, waist-to-hip ratio; male >0.9 defined as obese; female >0.85 defined as obese (*9, 10*).

3: BMI, body mass index (*10*).

4: The type of plaque, Small: psoriasis plaques are usually < 2 cm in diameter; Large: psoriasis plaques are usually ≥ 2 cm in diameter (*11*).

5: PASI, psoriasis area and severity index.

6: The improvement of PASI score (PASI%) = (the PASI score of psoriasis patients before the treatment of IL-17A inhibitor - the PASI score of psoriasis patients after the treatment of IL-17A inhibitor for 4 weeks)/the PASI score of psoriasis patients before the treatment of IL-17A inhibitor.

7: DLQI, dermatology life quality index.

8: The change of CD147 on CD4^+^ T cells (after treatment) (CD147_CD4^+^ T cells (%)) = (the expression of CD147 on the CD4^+^ T cells of psoriasis patients after the treatment of IL-17A inhibitor for 4 weeks- the expression of CD147 on the CD4^+^ T cells of psoriasis patients before the treatment of IL-17A inhibitor)/the expression of CD147 on the CD4^+^ T cells of psoriasis patients before the treatment of IL-17A inhibitor.

9: The change of CD147 on Th17 cells (after treatment) (CD147_Th17 cells (%)) = (the expression of CD147 on the Th17 cells of psoriasis patients after the treatment of IL-17A inhibitor for 4 weeks- the expression of CD147 on the Th17 cells of psoriasis patients before the treatment of IL-17A inhibitor)/the expression of CD147 on the Th17 cells of psoriasis patients before the treatment of IL-17A inhibitor.

**Reference**

1. P. Liu *et al.*, Acitretin Promotes the Differentiation of Myeloid-Derived Suppressor Cells in the Treatment of Psoriasis. *Front Med (Lausanne)* **8**, 625130 (2021).

2. K. Ghoreschi, A. Balato, C. Enerback, R. Sabat, Therapeutics targeting the IL-23 and IL-17 pathway in psoriasis. *Lancet* **397**, 754-766 (2021).

3. B. Kapoor, M. Gulati, P. Rani, R. Gupta, Psoriasis: Interplay between dysbiosis and host immune system. *Autoimmun Rev* **21**, 103169 (2022).

4. P. Liu *et al.*, The expression of mCTLA-4 in skin lesion inversely correlates with the severity of psoriasis. *J Dermatol Sci* **89**, 233-240 (2018).

5. R. Wu *et al.*, MicroRNA-210 overexpression promotes psoriasis-like inflammation by inducing Th1 and Th17 cell differentiation. *J Clin Invest* **128**, 2551-2568 (2018).

6. M. J. Pencina, R. B. D'Agostino, Overall C as a measure of discrimination in survival analysis: model specific population value and confidence interval estimation. *Stat Med* **23**, 2109-2123 (2004).

7. P. Liu *et al.*, Predicting the Risk of Psoriatic Arthritis in Plaque Psoriasis Patients: Development and Assessment of a New Predictive Nomogram. *Front Immunol* **12**, 740968 (2021).

8. K. Hu *et al.*, Should we customize the treatment with interleukin-17A inhibitors for moderate-to-severe psoriasis in the real-world setting? *J Eur Acad Dermatol Venereol*, (2022).

9. P. Song *et al.*, Temporal trends in normal weight central obesity and its associations with cardiometabolic risk among Chinese adults. *Sci Rep* **9**, 5411 (2019).

10. B. F. Zhou, C. Cooperative Meta-Analysis Group of the Working Group on Obesity in, Predictive values of body mass index and waist circumference for risk factors of certain related diseases in Chinese adults--study on optimal cut-off points of body mass index and waist circumference in Chinese adults. *Biomed Environ Sci* **15**, 83-96 (2002).

11. W. Lew, E. Lee, J. G. Krueger, Psoriasis genomics: analysis of proinflammatory (type 1) gene expression in large plaque (Western) and small plaque (Asian) psoriasis vulgaris. *Br J Dermatol* **150**, 668-676 (2004).
